# Genome-Wide Meta-Analysis of Cotinine Levels in Cigarette Smokers Identifies Locus at 4q13.2

**DOI:** 10.1038/srep20092

**Published:** 2016-02-01

**Authors:** Jennifer J. Ware, Xiangning Chen, Jacqueline Vink, Anu Loukola, Camelia Minica, Rene Pool, Yuri Milaneschi, Massimo Mangino, Cristina Menni, Jingchun Chen, Roseann E. Peterson, Kirsi Auro, Leo-Pekka Lyytikäinen, Juho Wedenoja, Alexander I. Stiby, Gibran Hemani, Gonneke Willemsen, Jouke Jan Hottenga, Tellervo Korhonen, Markku Heliövaara, Markus Perola, Richard J. Rose, Lavinia Paternoster, Nic Timpson, Catherine A. Wassenaar, Andy Z. X. Zhu, George Davey Smith, Olli T. Raitakari, Terho Lehtimäki, Mika Kähönen, Seppo Koskinen, Timothy Spector, Brenda W. J. H. Penninx, Veikko Salomaa, Dorret I. Boomsma, Rachel F. Tyndale, Jaakko Kaprio, Marcus R. Munafò

**Affiliations:** 1 MRC Integrative Epidemiology Unit (IEU) at the University of Bristol, Bristol, BS8 2BN, United Kingdom; 2 School of Social and Community Medicine, University of Bristol, Bristol, BS8 2BN, United Kingdom; 3 Virginia Institute for Psychiatric and Behavioral Genetics, Virginia Commonwealth University, Richmond, VA 23298, United States; 4 Department of Biological Psychology, VU University, Amsterdam, 1081 BT, Netherlands; 5 Department of Public Health, University of Helsinki, Helsinki, FI-00014, Finland; 6 Department of Psychiatry and EMGO Institute for Health and Care Research, VU University Medical Center, Amsterdam, 1081 HL, Netherlands; 7 Department of Twin Research and Genetic Epidemiology, King’s College London, London, SE1 7EH, United Kingdom; 8 National Institute for Health and Welfare, Helsinki, FI-00271, Finland; 9 Institute for Molecular Medicine, University of Helsinki, Helsinki, FI-00014, Finland; 10 Department of Clinical Chemistry, Fimlab Laboratories, Tampere, FI-33520, Finland; 11 Department of Clinical Chemistry, University of Tampere School of Medicine, Tampere, FI-33014, Finland; 12 University of Eastern Finland, Institute of Public Health and Clinical Nutrition, Kuopio, FI-70211, Finland; 13 Estonian Genome Center, University of Tartu, Tartu, 51010, Estonia; 14 Department of Psychological and Brain Sciences, Indiana University, Bloomington, IN 47405, United States; 15 Department of Pharmacology and Toxicology, University of Toronto, Toronto, M5S 1A8, Canada; 16 Department of Clinical Physiology and Nuclear Medicine, Turku University Hospital, Turku, FI-20521, Finland; 17 Research Centre of Applied and Preventive Cardiovascular Medicine, University of Turku, Turku, FI-20520, Finland; 18 Department of Clinical Physiology, Tampere University Hospital, Tampere, FI-33521, Finland; 19 Department of Clinical Physiology, University of Tampere School of Medicine, Tampere, FI-33014, Finland; 20 Department of Psychiatry, Campbell Family Mental Health Research Institute of the Centre for Addiction and Mental Health, University of Toronto, Toronto, Canada; 21 School of Experimental Psychology and UK Centre for Tobacco and Alcohol Studies, University of Bristol, Bristol, BS8 1TU, United Kingdom

## Abstract

Genome-wide association studies (GWAS) of complex behavioural phenotypes such as cigarette smoking typically employ self-report phenotypes. However, precise biomarker phenotypes may afford greater statistical power and identify novel variants. Here we report the results of a GWAS meta-analysis of levels of cotinine, the primary metabolite of nicotine, in 4,548 daily smokers of European ancestry. We identified a locus close to *UGT2B10* at 4q13.2 (minimum *p* = 5.89 × 10^−10^ for rs114612145), which was consequently replicated. This variant is in high linkage disequilibrium with a known functional variant in the *UGT2B10* gene which is associated with reduced nicotine and cotinine glucuronidation activity, but intriguingly is not associated with nicotine intake. Additionally, we observed association between multiple variants within the 15q25.1 region and cotinine levels, all located within the *CHRNA5-A3-B4* gene cluster or adjacent genes, consistent with previous much larger GWAS using self-report measures of smoking quantity. These results clearly illustrate the increase in power afforded by using precise biomarker measures in GWAS. Perhaps more importantly however, they also highlight that biomarkers do not always mark the phenotype of interest. The use of metabolite data as a proxy for environmental exposures should be carefully considered in the context of individual differences in metabolic pathways.

Genome-wide association studies (GWAS) have enjoyed considerable success in identifying genetic variants associated with complex behaviours such as cigarette smoking^1^,^2^,^3^,^4^. However, given the need for large sample sizes in GWAS, behavioural phenotypes are often assessed using self-report measures (e.g., number of cigarettes smoked per day). These may be subject to reporting biases (e.g., a smoker may report smoking less than he or she actually smokes) or error. Objective assessment of behavioural phenotypes, using a relevant biomarker, can address these limitations and provide greater measurement precision, therefore improving statistical power.

The rs16969968-rs1051730 single nucleotide polymorphism (SNP) within the *CHRNA5-A3-B4* gene cluster on chromosome 15 accounts for ~1% phenotypic variance in self-reported cigarettes per day, but ~4% phenotypic variance in cotinine levels (the primary metabolite of nicotine)^5^,^6^. It has previously been established that cigarette smokers modify their smoking behaviour to self-titrate nicotine to a level appropriate to individual need^7^,^8^. This may be achieved, for example, through timing of smoking in the day, varying number of puffs taken per cigarette, or volume of smoke inhaled per puff. Given individual differences in smoking topography, cotinine may provide a more accurate assessment of total tobacco exposure than cigarettes per day (self-reported or objectively assessed).

Previous, consortium-based GWAS of self-reported smoking quantity have identified a handful of independent genetic loci^1^,^2^,^3^,^4^. However, even these very large studies (n > 70,000) can only account for a small proportion of phenotypic variation. Using a biomarker phenotype may provide a valuable alternative approach given measurement precision and biological proximity. We therefore conducted a meta-analysis of genome-wide association data on cotinine levels in current, daily cigarette smokers, in order to identify genetic variants associated with smoking behaviour.

## Results

**Figure 1:**
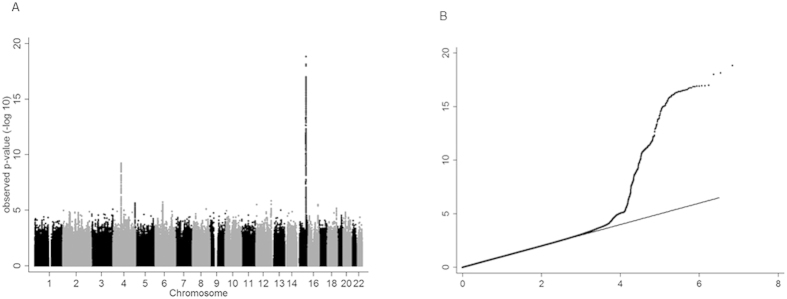
Manhattan and quantile-quantile plots illustrating genome-wide meta-analysis results. Manhattan plot (**A**): All SNPs plotted on *x*-axis according to their position on each chromosome, against their association with cotinine level, as shown on the *y*-axis as –log_10_ *p*-value. QQ plot (**B**): The observed distribution of *p*-values (*y*-axis) against the expected distribution of *p*-values under the null hypothesis (*x*-axis). Plots includes variants which were genotyped or imputed in at least 3,000 individuals only (~7 M SNPs).

**Figure 2:**
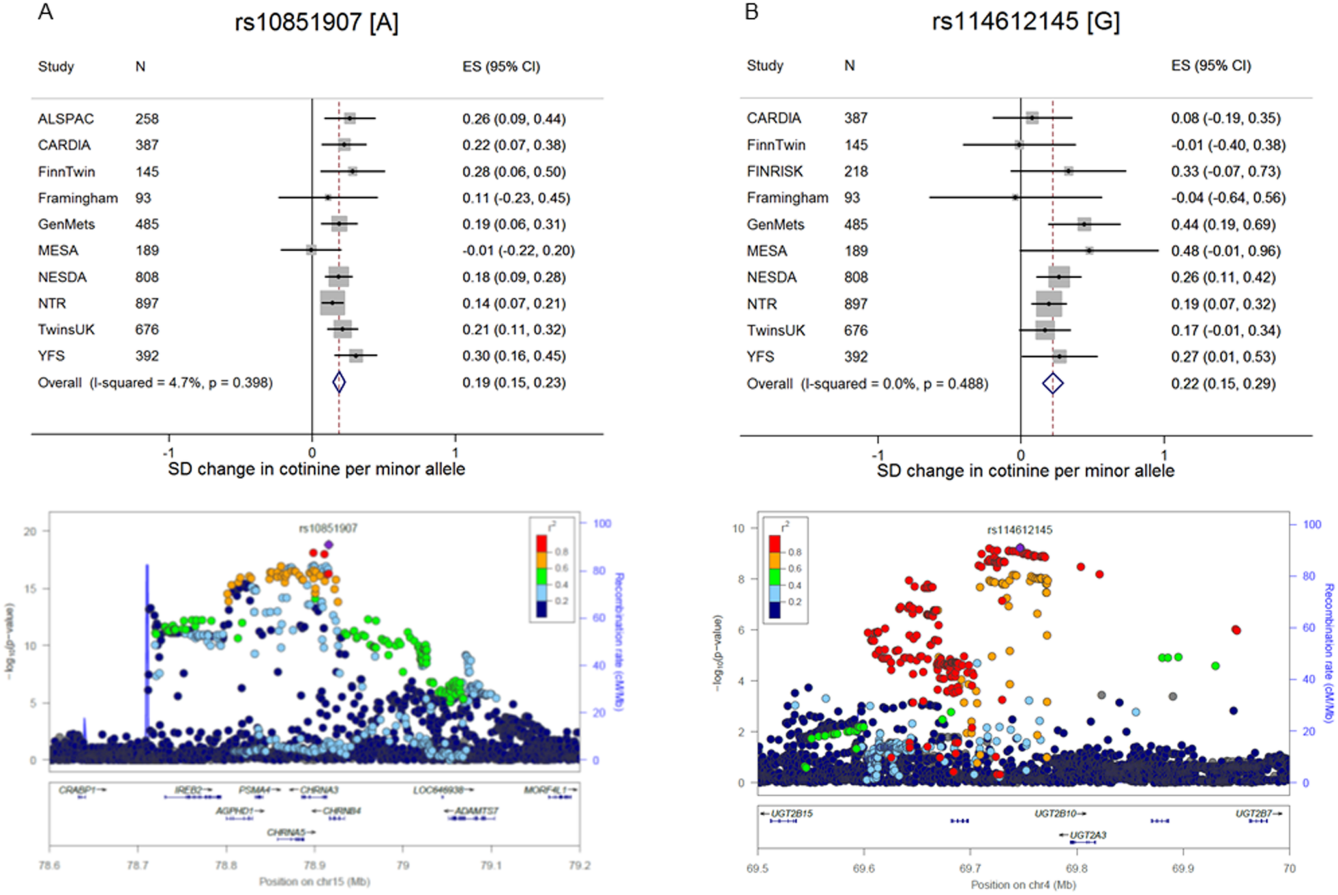
Forest and regional plots of associations for cotinine from genome-wide meta-analysis. Forest plots illustrate effect size and 95% confidence intervals (CIs) observed in each contributing study for chromosome 15 (**A**) and chromosome 4 (**B**) SNPs with smallest p-values (“top” SNPs). Regional plots show SNPs plotted by their positions on chromosomes against –log_10_ *p*-value for their association with cotinine level in genome-wide meta-analysis. The top SNP in each region is highlighted in purple. The SNPs surrounding each top SNP are colour coded to reflect their LD with this variant (see legend). Estimated recombination rates are plotted in pale blue to reflect local LD structure on secondary *y-*axis. Genome build = hg19; LD population = 1000 Genomes March 2012 release (EUR). Regional plots generated using Locus Zoom.

Variants in two genomic regions were found to be associated with cotinine levels, including 15q25.1 (a region previously identified in association with smoking quantity^1^,^3^,^4^) and a locus at 4q13.2 (see Figs 1 and 2, Table 1, Table S2). All 96 variants that met or exceeded the threshold for genome-wide significance (*p* < 5 × 10^−8^) on chromosome 4 lay between 69.6 and 69.9 Mb within a region of *UGT* genes, including *UGT2B10* and *UGT2A3* (see Fig. 2). The variant with the lowest *p*-value in this region was rs114612145 (rs77107237 in GRCh38) (*p* = 5.89 × 10^−10^), which lies between *UGT2B10* and *UGT2A3* (Table 1, Fig. 2). The minor allele (G) was associated with a 0.22 SD increase in cotinine level, equating to ~39 ng/ml increase in plasma/serum cotinine (Table 1, Fig. 2). The cotinine quantification method did not affect the result. This SNP accounted for 0.87% of the variance in cotinine levels.

To investigate whether the association with cotinine at 4q13.2 could be completely explained by rs114612145, we repeated the association analysis conditioning on this SNP (Figure S1, bottom panel). No residual signal was detected, suggesting that the variants identified in this region represent a single independent signal. For further confirmation of the cotinine association signal at 4q13.2, selected variants from this region were examined in relation to cotinine levels in two independent samples (see Tables S3 and S4). Strong evidence for association was observed in both (Table S4). The rs114612145 SNP identified in our sample is in high LD with a functional missense variant in *UGT2B10*, rs144647471 (also known as Asp67Tyr or rs61750900 in build GRCh38) (r^2^ = 0.90). Whilst this missense variant did not reach the threshold for genome-wide significance in our discovery sample (*p* = 1.91 × 10^−5^), this is likely due to the reduced sample size upon which association was determined (SNP imputed in only 2,585 individuals). However, we observed evidence of association in an independent sample, in the same direction to that observed in the discovery sample (*p* = 0.020; Table S3).

Our meta-analysis also highlighted a locus on chromosome 15, which has consistently been identified in GWAS of smoking quantity. All 279 genome-wide significant variants identified on this chromosome lay within the *CHRNA5-A3-B4* nicotinic receptor gene cluster, adjacent genes (*IREB2*, *AGPHD1*, *PSMA4* and *ADAMTS7*), or intergenic regions (see Fig. 2). The variant with the lowest *p*-value (both in this region and overall) was rs10851907 (*p* = 1.46 × 10^−19^), located in an intergenic region between *CHRNB4* and *CHRNA3* (Table 1, Fig. 2). The minor allele (A) was associated with a 0.19 SD increase in cotinine levels, equating to a ~34 ng/ml increase in plasma/serum cotinine (Table 1, Fig. 2). This SNP, which is in LD with rs16969968 (r^2^ = 0.71), accounted for 1.75% of the variance in cotinine levels.

To investigate whether the association with cotinine at 15q25.1 could be explained completely by rs10851907, we repeated the association analysis conditioning on this SNP (Figure S2). Residual association was detected within the region, with strongest evidence for association at rs57064725 (*p*_c_ = 2.92 × 10^−8^), located in an intron of *PSMA4* (Figure S2, middle panel). Conditioning on both of these variants left no residual signal (Figure S2, bottom panel). Given previous robust evidence linking measures of smoking quantity to the nonsynonymous SNP rs16969968, a variant which also exceeded the threshold for genome-wide significance in our analyses (*p* = 6.91 × 10^−17^), we also re-ran the association analysis conditioning on this SNP (Figure S3). Residual association similar to that observed after conditioning on rs10851907 was detected within the region, with strongest evidence for association at rs7170068 (*p*_c_ = 1.51 × 10^−9^), located in an intron in *CHRNA3* (Figure S3, middle panel). Conditioning on rs16969968 and rs7170068 left no residual signal in this region (Figure S3, bottom panel). It is notable that earlier dissection of the 15q25.1 region has suggested a third distinct signal for smoking quantity, marked by rs588765, apparent upon adjustment for rs16969968^9^,^10^. However, this signal was not identified in three previous large GWAS meta-analyses after conditioning on rs16969968^1^,^3^,^4^, and was not apparent in our data, before or after adjustment for rs16969968 (*p* = 0.10; *p*_c_ = 1.18 × 10^−3^).

## Discussion

We conducted a meta-analysis of genome-wide association data on cotinine levels in current smokers, in order to identify genetic variants associated with smoking behaviour. We identified 375 genetic variants, representing three independent signals. Two of these signals were located within *CHRNA5-A3-B4* on chromosome 15, a region that is now well-established as being associated with smoking heaviness. More importantly however, we also identified a signal within *UGT2B10* on chromosome 4, a locus which has previously been found to associate with cotinine and nicotine metabolism, but *not* with nicotine dose, highlighting an important limitation of the use of metabolite data (such as cotinine) as a proxy for an environmental exposure.

UGT2B10 plays a key role in nicotine and cotinine glucuronidation, converting nicotine to nicotine-glucuronide (a minor nicotine metabolic pathway, accounting for 3–5% nicotine clearance^11^,^12^,^13^), and cotinine to cotinine-glucuronide (accounting for 12–17% of cotinine clearance^11^). The minor allele at rs144647471 (i.e., that associated with higher cotinine levels in our sample) is associated with a reduction in UGT2B10 function for both nicotine and cotinine glucuronidation^14^,^15^.

Several factors contribute to cotinine levels: consumption of nicotine (i.e., heaviness of smoking), conversion of nicotine to cotinine (catalysed primarily by CYP2A6), and cotinine clearance (including cotinine glucuronidation). It is theoretically possible that the reduced function *UGT2B10* variant identified in this GWAS function could alter consumption. Reduced clearance of nicotine through glucuronidation (a minor pathway) would theoretically result in modestly longer lasting circulating levels of nicotine, which could *reduce* smoking if individuals self-titrate nicotine levels. Consistent with this theory, a previous study found that *ad libitum* smokers with this reduced function *UGT2B10* variant had lower nicotine intake^14^, as indexed by total nicotine equivalent levels. However, other studies (including a much larger study from the same group) have found that this variant has no effect on consumption^16^,^17^. Further, a decrease in nicotine intake via *ad libitum* smoking is *inconsistent* with higher cotinine levels, thus a change in consumption does not explain the association we note with the reduced function *UGT2B10* variant and higher cotinine levels. A more likely explanation for our findings is a reduction in cotinine metabolism, through decreased cotinine glucuronidation, which is consistent with the association noted between the reduced function *UGT2B10* variant and increased cotinine levels. Indeed, a recent GWAS conducted by Patel and colleagues^17^ concluded that genetic variation within *UGT2B10* contributes significantly to nicotine and cotinine glucuronidation but not to nicotine dose.

Cotinine is a strong biomarker of cigarette consumption in daily smokers^5^,^18^,^19^,^20^,^21^. The confirmation of the *CHRNA5-A3-B4* locus by our meta-analysis, and the conditional analyses results which parallel those of much larger GWAS of self-reported cigarette consumption^3^ demonstrate the utility of cotinine to identify genetic variants responsible for smoking quantity with greater statistical power (see also Table S5). However, since cotinine is an intermediate metabolite, its concentration is influenced by multiple contributing pathways. Individual differences in nicotine and cotinine metabolism, both via glucuronidation, as suggested here, and by oxidative metabolism as previously observed^18^ have confounding effects. While substantially more accurate than self-report smoking levels, future studies seeking an even more comprehensive assessment of tobacco exposure may consider using total nicotine equivalents (i.e., sum of nicotine and all its metabolites), a robust measure of nicotine exposure which intrinsically controls for variation in genes influencing both nicotine and cotinine metabolism (including variation in *UGT2B10* and *CYP2A6*)^12^,^22^.

In conclusion, we observed evidence of association between cotinine levels in current smokers and a locus at 4q13.2 encompassing *UGT2B10*, which encodes an enzyme playing a key role in nicotine and cotinine glucuronidation, in addition to the 15q25.1 region, previously shown to robustly associate with smoking quantity. Our analyses clearly illustrate the benefit of using precise, objective phenotypes in GWAS. However, they also importantly illustrate that biomarkers do not always capture the phenotype of interest. The use of metabolite data (such as cotinine) as a proxy for environmental exposures should be carefully considered in the context of individual differences in metabolic pathways.

## Methods

### Contributing studies

A total of 11 studies (collectively forming the Cotinine Consortium) contributed to the GWAS meta-analysis: Avon Longitudinal Study of Parents and Children (ALSPAC), Coronary Artery Risk Development in Young Adults (CARDIA), FinnTwin, FINRISK, Framingham Heart Study, Health2000 GenMets study (GenMets), Multi-Ethnic Study of Atherosclerosis (MESA), Netherlands Study of Depression and Anxiety (NESDA), Netherlands Twin Register (NTR), TwinsUK, and Cardiovascular Risk in Young Finns Study (YFS) (see Table 2). These 11 samples resulted in a collective sample size of *n* = 4,548. Further information on each study/cohort is provided in Text S1.

### Phenotype definition

Cotinine levels were determined from plasma, serum or urine samples, and quantified using immunoassay, radioimmunoassay or mass spectrometry (see Table 2 and Text S1 for further details). Cotinine levels show good agreement within samples across assessment methods, and further are highly correlated across different types of biological samples, including urine and blood^23^,^24^. However, given that absolute cotinine levels are much higher in urine relative to blood, cotinine data were transformed and standardised prior to the conduct of individual study-level GWAS analysis, to ensure meaningful comparison and synthesis of results across studies. Within each study sample, cotinine levels were assayed from one specific type of biological sample, using a singular assessment method.

### Sample inclusion criteria

Individuals within each sample were eligible for inclusion in analyses provided they were assessed for cotinine level at or after 17 years of age, of European ancestry, successfully genotyped genome-wide, and current *daily* smokers at the time of cotinine assessment. To minimise inclusion of non-smokers and non-daily smokers in our analyses, specific cotinine level inclusion thresholds were imposed, determined on the basis of receiver operating characteristic (ROC) analyses conducted in representative samples with smoking status self-report data and cotinine data derived from plasma and urine samples. These were conservatively set at 10 ng/ml cotinine in serum/plasma samples assessed using mass spectrometry, 50 ng/ml cotinine in serum/plasma samples assessed using immunoassay, and 80 ng/ml cotinine in urine samples assessed using immunoassay. Full descriptive characteristics of the studies in the Cotinine Consortium are presented in Table 2.

### Genotyping and imputation

All contributing studies performed their own genotyping, quality control, and imputation to the 1000 Genomes Phase 1 Version 3 reference panel (see Table S1). Study samples were genotyped on a number of different platforms. Each study applied its own set of quality control filters. Genotype imputation was performed using IMPUTE, IMPUTE2 or MACH prior to genome-wide association analyses.

### Study specific GWAS analysis

Prior to study-specific genome-wide association analyses, cotinine data were transformed if necessary to correct for positive skew (using natural logarithm or square-root), and then standardised (i.e., converted to Z-scores). An additive genetic model was used for association analyses. Linear regression was used to establish evidence of association, with standardised cotinine level as the dependent variable and allele dose (0, 1 or 2 copies of the minor allele) as the independent variable. All analyses adjusted for sex and age, with the exception of Framingham, which controlled only for sex as age data for this sample were not available. For family-based studies (e.g., NTR and FinnTwin), only one observation per family was included.

### Meta-analysis of GWAS results

All 11 GWAS summary data files were delivered to the co-ordinating site via secure file-sharing services. Imputation quality control procedures were centrally imposed. Specifically, variants were excluded if: a) MAF < 1%, and/or b) info score < 0.4 or r^2^ < 0.3. Once the quality of each data file was confirmed, files were imported into METAL (March 2011 Release) (http://www.sph.umich.edu/csg/abecasis/metal/index.html), a software tool for meta-analysis of whole genome association data. Genomic control was enabled (appropriate genomic control correction applied to input files) to correct for population structure. A fixed-effects meta-analysis was then performed for each SNP by combining allelic effects weighted by the inverse of their variance. Secondary correction for population structure via genomic control of summary statistics was not performed as the genomic control parameter (λGC) for meta-analysis summary statistics was 0.992. The fixed threshold for genome-wide significance was set at *p* < 5 × 10^−8^. The meta-analysis was completed for ~11 M variants. Results were limited to the ~7 M variants which had been genotyped/imputed in at least 3,000 individuals. The meta-analysis was also repeated using GWAMA 2.1^25^ assuming random effects and an additive model, and the same results were obtained.

### Conditional analyses

Conditional analyses were performed with GCTA software^26^ using full meta-analysis summary level statistics and a European reference panel (ALSPAC mothers cohort; n = 8,890) to determine the number of independent signals present in regions identified in our meta-analysis. For each region of interest, we re-ran the meta-analysis conditioning on our SNP with the lowest *p*-value. The next signal was identified from the conditional meta-analysis results, and included in the second conditional meta-analysis. This process was repeated in an iterative fashion until no residual signal remained below a threshold of *p* < 5 × 10^−8^ (see Figure S1 and S2). For 15q25.1, we also employed an additional strategy, conditioning on missense SNP rs16969968 first. The entire process was also repeated using an alternative sample (Netherlands Twin Register) as the LD reference panel (*n* = 7,000), and similar results obtained.

### Independent replication

We examined the association between the novel signal identified in our meta-analysis and cotinine levels in two independent samples, FINRISK2007 and FinnTwin (persons not included in discovery GWAS). Descriptive characteristics, genotyping and imputation information on these two samples is presented in Table S3.

## Acknowledgements

JJW is supported by a Post-Doctoral Research Fellowship from the Oak Foundation (http://www.oakfnd.org/). JJW, GH, LP, NT, GDS and MRM are members of the Medical Research Council (MRC) Integrative Epidemiology Unit: support from the MRC is gratefully acknowledged (MC_UU_12013/6). JJW and MRM are members of the UK Centre for Tobacco and Alcohol Studies, a UK Clinical Research Council Public Health Research: Centre of Excellence. Funding from British Heart Foundation, Cancer Research UK, Economic and Social Research Council, Medical Research Council, and the National Institute for Health Research, under the auspices of the UK Clinical Research Collaboration, is gratefully acknowledged. XC, JC and REP acknowledge support from the National Institute on Drug Abuse (NIDA) (R01DA032246). REP also acknowledges support from NIDA (R25 DA26119) and National Institute of Mental Health (NIMH) (T32 MH20030). AIS is supported by a Wellcome Trust 4-year PhD studentship in molecular, genetic, and lifecourse epidemiology (WT083431MA). LP is funded by a UK MRC Population Health Scientist fellowship (MR/J012165/1). VS was funded by the Academy of Finland (139635) and the Finnish Foundation for Cardiovascular Research. RJR acknowledges support from National Institute on Alcohol Abuse and Alcoholism (AA-12502). KA received sponsorship from the Finnish Medical Association. MP acknowledges EU FP7 Grants BBMRI-Large Prospective Cohorts (313010), and BioSHaRE (261433). YM is supported by GGZinGeest, the mental health care organization supporting NESDA. CM is supported by European Research Council (ERC) starting grant 284167 (P.I. JV). DB also acknowledges ERC support (230374). CW is supported by a Canadian Institutes of Health Research (CIHR) doctoral award. MM receives support from the National Institute for Health Research (NIHR) BioResource Clinical Research Facility and Biomedical Research Centre based at Guy’s and St Thomas’ NHS Foundation Trust and King’s College London. RFT is an Endowed Chair in Addiction for the Department of Psychiatry at the University of Toronto (CIHR grant TMH109787, NIH PGRN grant DA020830). JK is supported by Academy of Finland grants 265240 and 263278. Acknowledgements specific to individual cohorts are listed in Supplementary Information.

## Additional Information

**Data availability**: Cotinine GWAS meta-analysis summary results (doi: 10.5523/bris.182rhz19hg3lz1172a7yfcap9v) accessible in full at https://data.bris.ac.uk/data/dataset/182rhz19hg3lz1172a7yfcap9v.

**How to cite this article**: Ware, J. J. *et al.* Genome-Wide Meta-Analysis of Cotinine Levels in Cigarette Smokers Identifies Locus at 4q13.2. *Sci. Rep.* **6**, 20092; doi: 10.1038/srep20092 (2016).

## Supplementary Material

Supplementary Information

## Figures and Tables

**Table 1:** Summary information for top SNPs identified from cotinine genome-wide meta-analysis.

SNP	Chr	Gene	Position	EA	EAF	*n*	Beta	SE	*p* value
rs10851907	15	Intergenic (*CHRNB4*/*CHRNA3*)	78915864	A	0.41	4330	0.19	0.02	1.46 × 10^−19^
rs114612145	4	Intergenic (*UGT2B10*/*UGT2A3*)	69746647	G	0.10	4290	0.22	0.04	5.89 × 10^−10^

A total of 279 SNPs on chromosome 15 and 96 SNPs on chromosome 4 exceeded genome-wide significance for association with cotinine. The top SNP on each chromosome is shown. Position refers to base pair position in genome build hg19/GRCh37. EA: effect allele; EAF: effect allele frequency; SE: standard error; Beta: change in standard deviation of cotinine level per copy of the effect allele in an additive model.

**Table 2:** Descriptive characteristics of the 11 studies contributing to the genome-wide meta-analysis.

Study	*n*	Sex (% male)	Age (years)		Cotinine (ng/ml)^a^		Medium	Method
			Mean	SD	Mean	SD		
ALSPAC	258	50.4	17.8	0.4	182.1	107.2	Plasma	Immunoassay
CARDIA	387	47.8	25.3	3.4	202.0	137.8	Plasma	Radioimmunoassay
FinnTwin	145	46.2	23.0	1.5	206.6	107.5	Serum	Mass spectrometry
FINRISK	218	59.8	48.4	11.6	223.6	167.7	Serum	Mass spectrometry
Framingham	93	43.0	N/A	N/A	101.3	55.6	Plasma/serum	Mass spectrometry
GenMets	485	57.8	47.3	11.2	490.1	250.6	Serum	Immunoassay
MESA	189	57.5	59.6	8.9	4818.2	4105.5	Urine	Immunoassay
NESDA	808	36.5	41.5	12.4	260.9	224.4	Plasma	Immunoassay
NTR	897	44.1	43.6	13.9	278.5	269.7	Plasma	Immunoassay
TwinsUK	676	8.9	48.1	13.7	175.3	63.6	Plasma	Mass spectrometry
YFS	392	55.4	33.8	6.2	200.6	108.9	Serum	Mass spectrometry

^a^Cotinine mean and standard deviation values refer to raw values prior to standardisation (i.e., conversion to Z-scores). Further study details available in Text S1. SD: standard deviation.
