# Supplementary material for: Genome-Wide Meta-Analysis of Cotinine Levels in Cigarette Smokers Identifies Locus at 4q13.2
Source: Sci Rep. 2016 Feb 1;6:20092. doi: 10.1038/srep20092 (PMC4735517; doi:10.1038/srep20092)
Supplement: Supplementary Information [file srep20092-s1.pdf]

Genome-Wide Meta-Analysis of Cotinine Levels in Cigarette Smokers Identifies Locus at 4q13.2

Jennifer J. Ware, Xiangning Chen, Jacqueline Vink, Anu Loukola, Camelia Minica, Rene Pool, Yuri Milaneschi, Massimo Mangino, Cristina Menni, Jingchun Chen, Roseann Peterson, Kirsi Auro, Leo-Pekka Lyytikäinen, Juho Wedenoja, Alex I. Stiby, Gibran Hemani, Gonneke Willemsen, Jouke Jan Hottenga, Tellervo Korhonen, Markku Heliövaara, Markus Perola, Richard Rose, Lavinia Paternoster, Nic Timpson, Catherine A. Wassenaar, Andy Z. X. Zhu, George Davey Smith, Olli Raitakari, Terho Lehtimäki, Mika Kähönen, Seppo Koskinen, Timothy Spector, Brenda W. J. H. Penninx, Veikko Salomaa, Dorret Boomsma, Rachel F. Tyndale, Jaakko Kaprio, Marcus R. Munafò

Supplementary Information

Text S1: Cohort specific methods and acknowledgements

ALSPAC

Study description: The Avon Longitudinal Survey of Parents and Children (ALSPAC) is a prospective cohort study which recruited pregnant women residing in Avon, United Kingdom, with expected dates of delivery between 1 April 1991 and 31 December 1992. Full details of the study recruitment and methodology have been published previously [1]. A total of 14,541 pregnancies were included in the initial sample, resulting in 14,062 live births and 13,988 children who were alive at one year of age. Detailed information on mothers and their partners (during and after pregnancy) and the children (since birth) has been collected from self-report questionnaires and attendance at clinics. Our sample was limited to European offspring with available GWAS data and cotinine data (collected at age 17). Ethics approval

for the study was obtained from the ALSPAC Ethics and Law Committee and the Local Research Ethics Committee. Please note that the study website contains details of all the data that is available through a fully searchable data dictionary (http://www.bristol.ac.uk/alspac/researchers/data-access/data-dictionary/).

Cotinine assessment: Cotinine was assayed from ethylenediaminetetraacetic acid serum plasma samples taken in a clinical assessment at 17 years. Plasma samples were stored at −80 °C and allowed to thaw at room temperature before use. Cotinine was measured using the Cozart Cotinine Enzyme Immunoassay (Concateno, [now Alere Toxicology] UK, Abingdon) serum kit (M155B1). All samples, calibrators, and controls were brought to room temperature before use and were run in duplicate. Where required, samples were diluted using cotinine-free serum (fetal calf serum). Absorbance was measured spectrophotometrically at a wavelength of 450nm. The lowest calibrator used was 0.5ng/ml serum, and values below this were treated as undetectable/null (0ng/ml serum).

Acknowledgements: We are extremely grateful to all the families who took part in this study, the midwives for their help in recruiting them, and the whole ALSPAC team, which includes interviewers, computer and laboratory technicians, clerical workers, research scientists, volunteers, managers, receptionists and nurses. The UK Medical Research Council and the Wellcome Trust (Grant ref: 092731) and the University of Bristol provide core support for ALSPAC. ALSPAC GWAS data were generated by Sample Logistics and Genotyping Facilities at the Wellcome Trust Sanger Institute and LabCorp (Laboratory Corportation of America) using support from 23andMe.

CARDIA

Study description: CARDIA was designed to examine the development, determinants, and risk factors of clinical and subclinical cardiovascular disease. Participants were a part of The

National Heart, Lung, and Blood Institute's Coronary Artery Risk Development in Young Adults 'CARDIA' Study. A total of 5,115 young adult African and European American men and women completed the baseline examination in 1985–1986. The participants were selected so that there would be approximately the same number of people in subgroups of race, gender, education (high school or less and more than high school), and age (18–24 and 25–30) in each of four centers: Birmingham, AL; Chicago, IL; Minneapolis, MN; and Oakland, CA. Periodic follow-up examinations were held through 2006 with high participant retention (72–90%). Additional details can be found on the CARDIA website (http://www.cardia.dopm.uab.edu/). Some of the self-reported current smokers had base-line serum cotinine levels determined. Our study used the data from those subjects with cotinine assessment and of European ancestry.

Cotinine assessment: Blood for cotinine level analysis was collected under a standardized protocol. A 1ml aliquot of serum was frozen and shipped to the Clinical Biochemistry Laboratories at the American Health Foundation. Cotinine was determined by radioimmunoassay using the method of Haley et al. [2] after a modification of the method described by VanVunakis et al [3 4].

Acknowledgements: The Coronary Artery Risk Development in Young Adults Study (CARDIA) is conducted and supported by the National Heart, Lung, and Blood Institute (NHLBI) in collaboration with the University of Alabama at Birmingham (N01-HC95095 & N01-HC48047), University of Minnesota (N01-HC48048), Northwestern University (N01-HC48049), and Kaiser Foundation Research Institute (N01-HC48050). This manuscript was not approved by CARDIA. The opinions and conclusions contained in this publication are solely those of the authors, and are not endorsed by CARDIA or the NHLBI and should not be assumed to reflect the opinions or conclusions of either. Funding for CARe genotyping was provided by NHLBI Contract N01-HC-65226. Genotyping for the CARDIA GENEVA

cohort was supported by grant U01 HG004729 from the National Human Genome Research Institute.

FinnTwin12 and FinnTwin16

Study description: Analyses were based on samples from unrelated individuals from the FinnTwin12 (FT12) and FinnTwin16 (FT16) cohort studies. FT12 is a population based-cohort longitudinal study of five consecutive birth cohorts of Finnish twins born between 1983 and 1987. All twins were initially contacted and invited to participate by mail in the autumn of the year in which their birth cohort reached 11 years of age, with follow-ups when the twins were aged 14, 17 and approximately 22 years. The FT16 is a population-based longitudinal study of five consecutive birth cohorts of Finnish twins born between 1975 and 1979. Each pair was initially approached immediately after the twins' 16th birthday. The baseline data collection started in 1991 and was completed in 1996. Waves 2-5 were made when the twins were 17, 18.5, approximately 25 years, and most recently at an average age of 34 years. For both the FT12 and FT16, the baseline and follow-up assessments included surveys of smoking habits and other health-related variables. In addition, in wave 4 blood samples for DNA extraction and biochemistry analyses were taken from all twins during a visit to the twin research clinic in Helsinki (Finland). Altogether 145 subjects were included in the GWAS discovery phase, with genome-wide genotype data generated with the Illumina Human670-QuadCustom BeadChip (Illumina, Inc., San Diego, CA, USA). In the replication phase 135 additional subjects were included, with genotype data for the top loci extracted from genome-wide genotype data generated with the Illumina HumanCoreExome-12v1-0 BeadChip and imputed according to the same protocol as for the discovery sample.
Data collection and analysis were approved by the ethics committees of the Department of Public Health of the University of Helsinki, the Helsinki and Uusimaa Hospital District and

the IRB of Indiana University. Written informed consent was obtained from all participating twins.

Cotinine assessment: All biological samples were stored at -80°C at the National Institute for Health and Welfare. Serum samples were sent for cotinine measurement to University of Toronto (Dr. Rachel Tyndale). Samples were assessed by LCMS as previously described [5].

Acknowledgements: The study has been supported by ENGAGE – European Network for Genetic and Genomic Epidemiology, FP7-HEALTH-F4-2007, grant agreement number 201413, Academy of Finland (grant numbers 265240 and 263278), and the Sigrid Juselius Foundation (to JK). Genome-wide genotyping was funded by Global Research Award for Nicotine Dependence/Pfizer Inc. (to JK), NIH (AA15416 to DM Dick), and Wellcome Trust Sanger Institute, UK. Phenotype collection has been supported by NIAAA (AA-12502, AA-00145, and AA-09203) and the Academy of Finland (141054 and 264146 to JK). Antti-Pekka Sarin and Samuli Ripatti are acknowledged for genotype imputation and post-imputation quality controls. Cotinine assessment was supported by the Canadian Institutes of Health Research (TMH109787) and NIH (PGRN DA020830) to Rachel Tyndale.

FINRISK 1992 and FINRISK2007

Study description: The National FINRISK Study monitors risk factor level trends every five years since 1972. In 1992, the FINRISK survey was carried out to assess cardiovascular risk factor levels in Finland. The survey was conducted in four areas of Finland: (1) North Karelia province, (2) Kuopio, (3) Turku and Loimaa (representing South western Finland), and (4) Helsinki and Vantaa. A random sample of approximately 2,000 individuals aged 25-64 from each of the survey areas was drawn from the National Population Register. Altogether 7,927 individuals were enrolled, and 6,051 participated (76.3% participation rate). The survey

included a self-administered questionnaire (mainly covering questions on socioeconomic factors, medical history, health behavior, and psychological factors) and a cardiovascular risk factor examination. A detailed smoking history as described by Vartiainen and colleagues[6] was obtained. Blood samples for DNA extraction and biochemistry analyses were taken as part of the risk factor examination.

In 2007, the FINRISK survey was carried out in six regions of Finland: 1) Helsinki and Vantaa, 2) Turku and Loimaa, 3) North Savo, 4) North Karelia, 5) the Oulu region, and 6) Lapland. A two stage process was used. Participants (n = 11,953) from all regions were invited to fill in an extensive baseline questionnaire (n = 7,993, 67% response rate) and to attend a locally organized health examination in which blood samples were taken (all regions but Lapland). After the baseline study, a self-administered questionnaire with detailed smoking history was given to individuals who had stated during the first part of the study that they had smoked at least 100 cigarettes during their lifetime (regions 1-3), or that they were current smokers (regions 4 and 5) (n = 1,992). Completed questionnaires were returned by mail, with one reminder. The number of participants in the smoking sub-study was 1,746 (91% response rate). Plasma cotinine was analyzed for those who identified themselves as daily smokers during the main FINRISK data collection and responded to the tobacco-specific questionnaire[7].

Altogether 218 subjects from FINRISK 1992 and FINRISK 2007 were included in the GWAS discovery phase, with genome-wide genotype data generated with the Illumina HumanOmniExpress BeadChip (Illumina, Inc., San Diego, CA, USA). These samples belong to the Predict-CVD sub-cohort, which is a random subset of the whole FINRISK cohort, and has been previously described by Ganna and colleagues[8]. The Predict CVD -sample is enriched for individuals with a diagnosed cardiovascular event (coronary heart disease and/or ischemic stroke). The participants originate from FINRISK cohorts collected on 1992, 1997,

2002 and 2007. The size of sub-cohort in each stratum was made proportional to the number of incident cardiovascular disease cases in the corresponding stratum.

In the replication phase 620 additional subjects from FINRISK 2007 were included, with genotype data for the top loci extracted from genotype data generated with the MetaboChip. The MetaboChip is a custom Illumina iSelect genotyping array designed to test ~200,000 SNPs of interest for metabolic and atherosclerotic / cardiovascular disease traits. Content on the chip was selected on the basis of large scale meta-analysis of relevant traits (including up to 100,000 individuals) and of HapMap and 1000 Genomes Project SNP content. Due to the sparse SNP coverage of the MetaboChip, no imputation was performed. These samples belong to the DILGOM sample, which is an extension of the FINRISK 2007 survey consisting of individuals of 25-74 years age who participated in the Dietary, Lifestyle and Genetic determinants of Obesity and Metabolic syndrome study which was carried out in Helsinki/Vantaa region in Southern Finland.

The FINRISK studies were approved by the Ethical Committee of the Hospital District of Helsinki and Uusimaa. All the surveys were conducted according to the ethical rules of the National Public Health Institute, and the investigations were performed in accordance with the Declaration of Helsinki. All participants gave their written informed consent prior to participation in the study.

Cotinine assessment: Plasma cotinine from fasting blood samples was analysed with a gas chromatograph-mass spectrometer (GC-MS) at the National Institute for Health and Welfare, Helsinki, Finland as previously described in detail [6, 7].

Acknowledgements: FINRISK has been primarily funded by budgetary funds of THL (National Institute for Health and Welfare). Important additional funding has been obtained from the Academy of Finland (grant number 139635 for VS) and from the Finnish

Foundation for Cardiovascular Research. Antti-Pekka Sarin and Samuli Ripatti are acknowledged for genotype imputation and post-imputation quality controls.

Framingham

Study description: In 1948, the researchers recruited 5,209 men and women between the ages of 30 and 62 from the town of Framingham, Massachusetts, and began the first round of extensive physical examinations and lifestyle interviews that they would later analyze for common patterns related to CVD development. Since 1948, the subjects have returned to the study every two years for an examination consisting of a detailed medical history, physical examination, and laboratory tests, and in 1971, the study enrolled a second-generation cohort -- 5,124 of the original participants' adult children and their spouses -- to participate in similar examinations. The second examination of the Offspring cohort occurred eight years after the first examination, and subsequent examinations have occurred approximately every four years thereafter. In April 2002 the Study entered a new phase: the enrolment of a third generation of participants, the grandchildren of the original cohort. The first examination of the Third Generation Study was completed in July 2005 and involved 4,095 participants. Thus, the FHS has evolved into a prospective, community-based, three generation family study. The FHS is a joint project of the National Heart, Lung and Blood Institute and Boston University. Part of the FHS subjects participated in a plasma metabolite sub-study. Data used in our study were extracted from this metabolite profiling study.

Cotinine assessment: Cotinine was assayed from plasma samples using liquid chromatography-tandem mass spectrometry (LC-MS) with a 4000 QTRAP triple quadrupole mass spectrometer (Applied Biosystems/Sciex) that was coupled to a multiplexed LC system comprised of two 1200 Series pumps (Agilent Technologies) and an HTS PAL autosampler (Leap Technologies) equipped with two injection ports and a column selection valve [9].

Acknowledgements: The Framingham Heart Study is conducted and supported by the National Heart, Lung, and Blood Institute (NHLBI) in collaboration with Boston University (Contract No. N01-HC-25195). This manuscript was not prepared in collaboration with investigators of the Framingham Heart Study and does not necessarily reflect the opinions or views of the Framingham Heart Study, Boston University, or NHLBI. Funding for SHARe Affymetrix genotyping was provided by NHLBI Contract N02-HL-64278. SHARe Illumina genotyping was provided under an agreement between Illumina and Boston University. Funding support for the Framingham Metabolomics (HILIC - Installment 2) dataset was provided by NIH grant R01 DK081572.

GenMets/Health2000

Study description: The Health2000 study is a nationally representative sample of adult Finnish population, which includes a total of 8,028 subjects aged 30 or over. They were invited for an in-person study during which a blood sample was taken for cotinine measures and for DNA extraction. A detailed smoking history as described by Keskitalo and colleagues [10] was obtained. Altogether 485 subjects from GenMets were included in the GWAS discovery phase, with genome-wide genotype data generated with the Human610-Quad BeadChip (Illumina, Inc., San Diego, CA, USA). All participants gave written informed consent, and the study was approved by the Ethics Committee for Epidemiology and Public Health of the Hospital District of Helsinki and Uusimaa, Finland.

Cotinine assessment: The cotinine concentration (ng/ml) was determined from the serum using liquid-phase radioimmunoassay methodology (Nicotine Metabolite DOUBLE ANTIBODY kit, Diagnostic Products Corporation, Los Angeles, CA, USA).

Acknowledgements: GENMETS is a subsample of the Health 2000 Study. The Health 2000 Study is funded by the National Institute for Health and Welfare (THL), the Finnish Centre

for Pensions (ETK), The Social Insurance Institution of Finland (KELA), The Local Government Pensions Institution (KEVA) and other organizations listed on the website of the survey (http://www.terveys2000.fi). GWAS genotyping was supported by the Wellcome Trust Sanger Institute. Antti-Pekka Sarin and Samuli Ripatti are acknowledged for genotype imputation and post-imputation quality controls.

MESA

Study description: The MESA (Multi-Ethnic Study of Atherosclerosis) [11] is a multicenter prospective cohort study to investigate the prevalence, correlates, and progression of subclinical cardiovascular disease in persons without clinical cardiovascular disease. In 2000 to 2002, MESA recruited 6,814 men and women aged 45 to 84 years from 6 U.S. communities: Forsyth County, North Carolina; northern Manhattan and Bronx, New York; Baltimore and Baltimore County, Maryland; St. Paul, Minnesota; Chicago, Illinois; and Los Angeles, California. Exclusion criteria included clinical cardiovascular disease, body weight greater than 300 lb, pregnancy, or impediment to long-term participation.

Cotinine assessment: Cotinine was assayed from urine samples using immunoassay (Immulite 2000 Nicotine Metabolite Assay, Diagnostic Products, Los Angeles, California) at the National Institute for Occupational Safety and Health Core Laboratory. The minimum detectable concentration was 10 ng/ml.

Acknowledgements: MESA and the MESA SHARe project are conducted and supported by the National Heart, Lung, and Blood Institute (NHLBI) in collaboration with MESA investigators. Support for MESA is provided by contracts N01-HC- 95159, N01-HC-95160, N01-HC-95161, N01-HC-95162, N01-HC-95163, N01-HC-95164, N01-HC-95165, N01-HC-95166, N01-HC-95167, N01-HC-95168, N01-HC-95169 and CTSA UL1-RR 024156. Funding for SHARe genotyping was provided by NHLBI Contract N02-HL-64278.

Genotyping was performed at Affymetrix (Santa Clara, California, USA) and the Broad Institute of Harvard and MIT (Boston, Massachusetts, USA) using the Affymetric Genome-Wide Human SNP Array 6.0. Funding support for the Lung CT dataset was provided by grant HL077612-05A1.

NESDA

Study description: The Netherlands Study of Depression and Anxiety (NESDA) is an ongoing cohort study into the long-term course and consequences of depressive and anxiety disorders. A description of the study rationale, design and methods is given elsewhere [12]. Briefly, in 2004 2,007 participants aged 18–65 years were recruited from the community (19%), general practice (54%) and secondary mental health care (27%). A total of 2,981 participants were included, consisting of persons with a current or past depressive and/or anxiety disorder and healthy controls. The research protocol was approved by the ethical committee of participating universities, and all respondents provided written informed consent.

Cotinine assessment: Fasting blood samples of NESDA participants were collected in each of the participating centers and kept frozen at -80 Celsius degrees. Cotinine concentrations were determined in the laboratory (Good Biomarker Sciences, Leiden, the Netherlands), and were assessed in blood plasma by solid phase competitive ELISA (Cotinine Direct ELISA kit, cat. no: CO096D, Calbiotech, CA, USA) according to the manufacturer's instructions. The detection limit was 1 ng/ml. Intra- and inter-assay coefficients of variation for values >2 ng/ml were >20% and >15%, respectively.

Acknowledgements: Funding was obtained from the Netherlands Organization for Scientific Research (Geestkracht program grant 10-000-1002); the Center for Medical Systems Biology

(CSMB, NWO Genomics), Biobanking and Biomolecular Resources Research Infrastructure (BBMRI-NL), VU University's Institutes for Health and Care Research (EMGO+) and Neuroscience Campus Amsterdam, University Medical Center Groningen, Leiden University Medical Center, National Institutes of Health (NIH, R01D0042157-01A, MH081802, Grand Opportunity grants 1RC2 MH089951 and 1RC2 MH089995). Part of the genotyping and analyses were funded by the Genetic Association Information Network (GAIN) of the Foundation for the National Institutes of Health. Computing was supported by BiG Grid, the Dutch e-Science Grid, which is financially supported by NWO.

NTR

Study description: The Netherlands Twin Register (NTR) includes a longitudinal study of health, medication use, addiction, lifestyle and personality among adolescent and adult participants. Since 1986, data were collected every two years in more than 33,000 twins and their family members, i.e., siblings, spouses, parents and offspring of twins [13, 14, 15, 16]. The study protocol was approved by Central Ethics Committee on Research Involving Human Subjects of the VU University Medical Center, Amsterdam, an Institutional Review Board certified by the US Office of Human Research Protections (IRB number IRB-2991 under Federal-wide Assurance-3703; IRB/institute codes, NTR 03-180). All participants provided informed consent.

Cotinine assessment: Cotinine was assessed in blood plasma. Fasting blood samples were collected during a home visit between 7:00 and 10:00 AM and stored at −27 °C. Cotinine assessment was carried out by using the Cotinine Direct ELISA kit, (cat.no: CO096D, Calbiotech, CA, USA) following manufacturer's instructions. For cotinine values in the 2-10 ng/ml range we used a threshold of 20% for the inter-assay coefficient of variation, whereas

for values exceeding 10 ng/ml the threshold was 15%. Values below 1 ng/ml were treated as undetectable.

Acknowledgements: We thank the Netherlands Twin Register participants whose data were analyzed in this study. We acknowledge the Netherlands Organisation for Scientific Research (NWO) and the Netherlands Organisation for Health Research and Development (ZonMW), the EMGO+ Institute for Health and Care Research, the Neuroscience Campus Amsterdam, BBMRI – NL (184.021.007: Biobanking and Biomolecular Resources Research Infrastructure), and the European Research Council (230374, 284167) for support. Genotyping was funded in part by grants from the National Institutes of Health (4R37DA018673-06, RC2 MH089951).

TwinsUK

Study description: Study subjects were twins enrolled in the TwinsUK registry, a national register of adult twins. Twins were recruited as volunteers by successive media campaigns without selecting for particular diseases or traits[17]. In this study we analysed data from 674 female twins who cotinine data available. The study was approved by St. Thomas' Hospital Research Ethics Committee, and all twins provided informed written consent.

Cotinine assessment: Cotinine levels in TwinsUK were measured using a non-targeted mass spec-based metabolomic profiling using the Metabolon platform, as described previously[18]. Metabolomic profiling was done in three batches. The raw cotinine levels were median-normalised (dividing each cotinine concentration by the day cotinine median), then inverse normalised as the metabolite concentration was not normally distributed. To measure the correlation between Metabolon platform and LCMS method described in [5], 12 samples were analysed with both methods.

Acknowledgements: TwinsUK was funded by the Wellcome Trust; European Community's Seventh Framework Programme (FP7/2007-2013). The study also receives support from the National Institute for Health Research (NIHR) BioResource Clinical Research Facility and Biomedical Research Centre based at Guy's and St Thomas' NHS Foundation Trust and King's College London. SNP Genotyping was performed by The Wellcome Trust Sanger Institute and National Eye Institute via NIH/CIDR.

YFS

Study description: The Cardiovascular Risk in Young Finns Study (YFS)[19] is a 5-center follow-up study of atherosclerosis risk factors in Finnish children and adolescents (http://med.utu.fi/cardio/youngfinnsstudy/). The first cross-sectional survey was conducted in 1980. Altogether 4,320 children and adolescents aged 3, 6, 9, 12, 15, and 18 years, randomly chosen from the national register, were enrolled. There were 3,596 participants (83.2% participation rate) in 1980. Follow-up studies were conducted 3 years apart, in 1983 and 1986. In 2001, we re-examined these individuals, who had then reached the age of 24 to 39 years. In 2001, genomic DNA was extracted from peripheral blood leukocytes. The loss of participants to follow-up was approximately 20%, 30%, and 34% after 3, 6, and 21 years, respectively. In adulthood, the latest 27-year follow-up study was conducted in 2007 (ages 30-45 years) with 2,204 participants. Smoking habits were determined using a questionnaire in participants aged 21 years or older. In 12- to 18-year-olds, the information on smoking habits was collected in connection with the medical examination. The study was approved by the local ethics committees (the University Hospitals of Helsinki, Turku, Tampere, Kuopio and Oulu) and was conducted following the guidelines of the Declaration of Helsinki. All participants gave their written informed consent.

Cotinine assessment: Serum samples were sent for cotinine measurement to University of Toronto (Dr. Rachel Tyndale). Samples were assessed by LCMS as previously described [5].

Acknowledgements: The Young Finns Study has been financially supported by the Academy of Finland: grants 134309 (Eye), 126925, 121584, 124282, 129378 (Salve), 117787 (Gendi), and 41071 (Skidi), the Social Insurance Institution of Finland, Kuopio, Tampere and Turku University Hospital Medical Funds (grant 9M048 and 9N035 for T. Lehtimäki), Juho Vainio Foundation, Paavo Nurmi Foundation, Finnish Foundation of Cardiovascular Research and Finnish Cultural Foundation, Tampere Tuberculosis Foundation and Emil Aaltonen Foundation (for T. Lehtimäki). The expert technical assistance in the statistical analyses by Ville Aalto and Irina Lisinen is gratefully acknowledged. Antti-Pekka Sarin and Samuli Ripatti are acknowledged for genotype imputation and post-imputation quality controls. Cotinine assessment was supported by the Canadian Institutes of Health Research (TMH109787) and NIH (PGRN DA020830) to Rachel Tyndale.

References

1. Boyd A*, et al.* Cohort Profile: the 'children of the 90s'--the index offspring of the Avon Longitudinal Study of Parents and Children. *International journal of epidemiology* **42**, 111-127 (2013).

2. Haley NJ, Axelrad CM, Tilton KA. Validation of self-reported smoking behavior: biochemical analyses of cotinine and thiocyanate. *American journal of public health* **73**, 1204-1207 (1983).

3. Van Vunakis H, Gjika HB, Langone JJ. Radioimmunoassay for nicotine and cotinine. *IARC scientific publications*, 317-330 (1987).

4. Hamidovic A*, et al.* Gene-centric analysis of serum cotinine levels in African and European American populations. *Neuropsychopharmacology : official publication of the American College of Neuropsychopharmacology* **37**, 968-974 (2012).

5. St Helen G*, et al.* Reproducibility of the nicotine metabolite ratio in cigarette smokers. *Cancer epidemiology, biomarkers & prevention : a publication of the American Association for Cancer Research, cosponsored by the American Society of Preventive Oncology* **21**, 1105-1114 (2012).

6. Vartiainen E, Seppala T, Lillsunde P, Puska P. Validation of self reported smoking by serum cotinine measurement in a community-based study. *Journal of epidemiology and community health* **56**, 167-170 (2002).

7. Broms U*, et al.* Diurnal Evening Type is Associated with Current Smoking, Nicotine Dependence and Nicotine Intake in the Population Based National FINRISK 2007 Study. *Journal of addiction research & therapy* **S2**, (2012).

8. Ganna A*, et al.* Utilizing twins as controls for non-twin case-materials in genome wide association studies. *PloS one* **8**, e83101 (2013).

9. Wang TJ*, et al.* Metabolite profiles and the risk of developing diabetes. *Nature medicine* **17**, 448-453 (2011).

10. Keskitalo K*, et al.* Association of serum cotinine level with a cluster of three nicotinic acetylcholine receptor genes (CHRNA3/CHRNA5/CHRNB4) on chromosome 15. *Human molecular genetics* **18**, 4007-4012 (2009).

11. Bild DE*, et al.* Multi-ethnic study of atherosclerosis: objectives and design. *American journal of epidemiology* **156**, 871-881 (2002).

12. Penninx BW, *et al.* The Netherlands Study of Depression and Anxiety (NESDA): rationale, objectives and methods. *International journal of methods in psychiatric research* **17**, 121-140 (2008).

13. Boomsma DI, *et al.* Netherlands Twin Register: from twins to twin families. *Twin Research and Human Genetics* **9**, 849-857 (2006).

14. Boomsma DI, *et al.* Netherlands Twin Register: a focus on longitudinal research. *Twin Research* **5**, 401-406 (2002).

15. Willemsen G, *et al.* The Netherlands Twin Register biobank: a resource for genetic epidemiological studies. *Twin Research and Human Genetics* **13**, 231-245 (2010).

16. Willemsen G, *et al.* The Adult Netherlands Twin Register: twenty-five years of survey and biological data collection. *Twin Research and Human Genetics* **16**, 271-281 (2013).

17. Moayyeri A, Hammond CJ, Valdes AM, Spector TD. Cohort Profile: TwinsUK and healthy ageing twin study. *International journal of epidemiology* **42**, 76-85 (2013).

18. Shin SY, *et al.* An atlas of genetic influences on human blood metabolites. *Nature genetics* **46**, 543-550 (2014).

19. Raitakari OT, *et al*. Cohort profile: the cardiovascular risk in Young Finns Study. *International journal of epidemiology* **37**, 1220-1226 (2008).

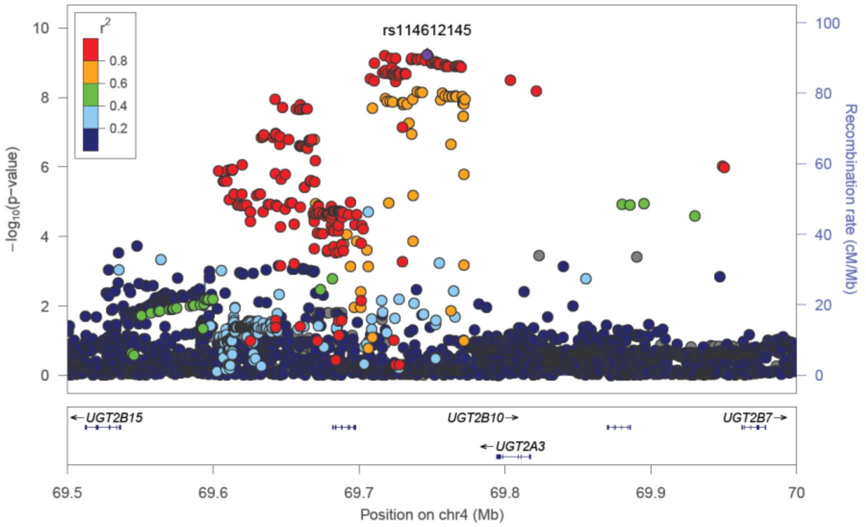

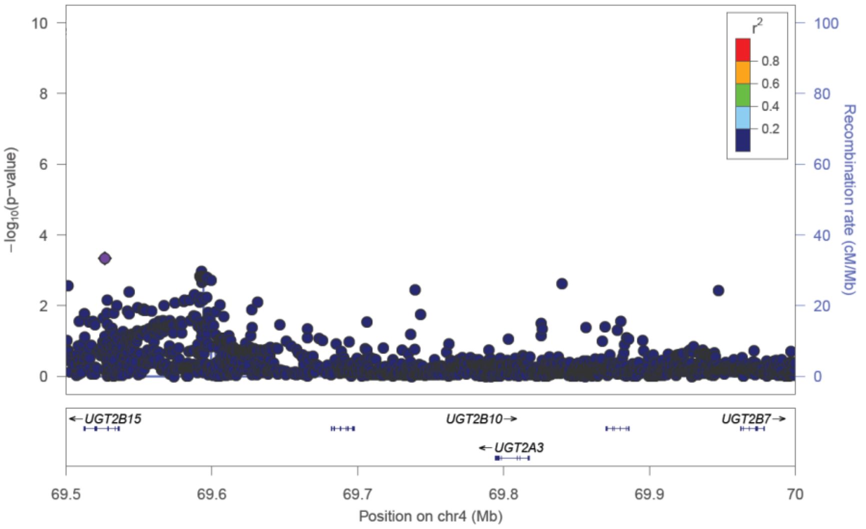

Figure S1. Chromosome 4 conditional analyses. Panels illustrate regional association plots of 4q13.2 region (69.5 to 70MB) based on the original results (top), and after conditioning on top hit rs114612145 (bottom). SNPs plotted by their position on chromosome 4 against $-\log_{10}$ *p*-value for their association with cotinine level in genome-wide meta-analysis. The ALSPAC mothers cohort (n=8,890) was used as an LD reference panel for these analyses.

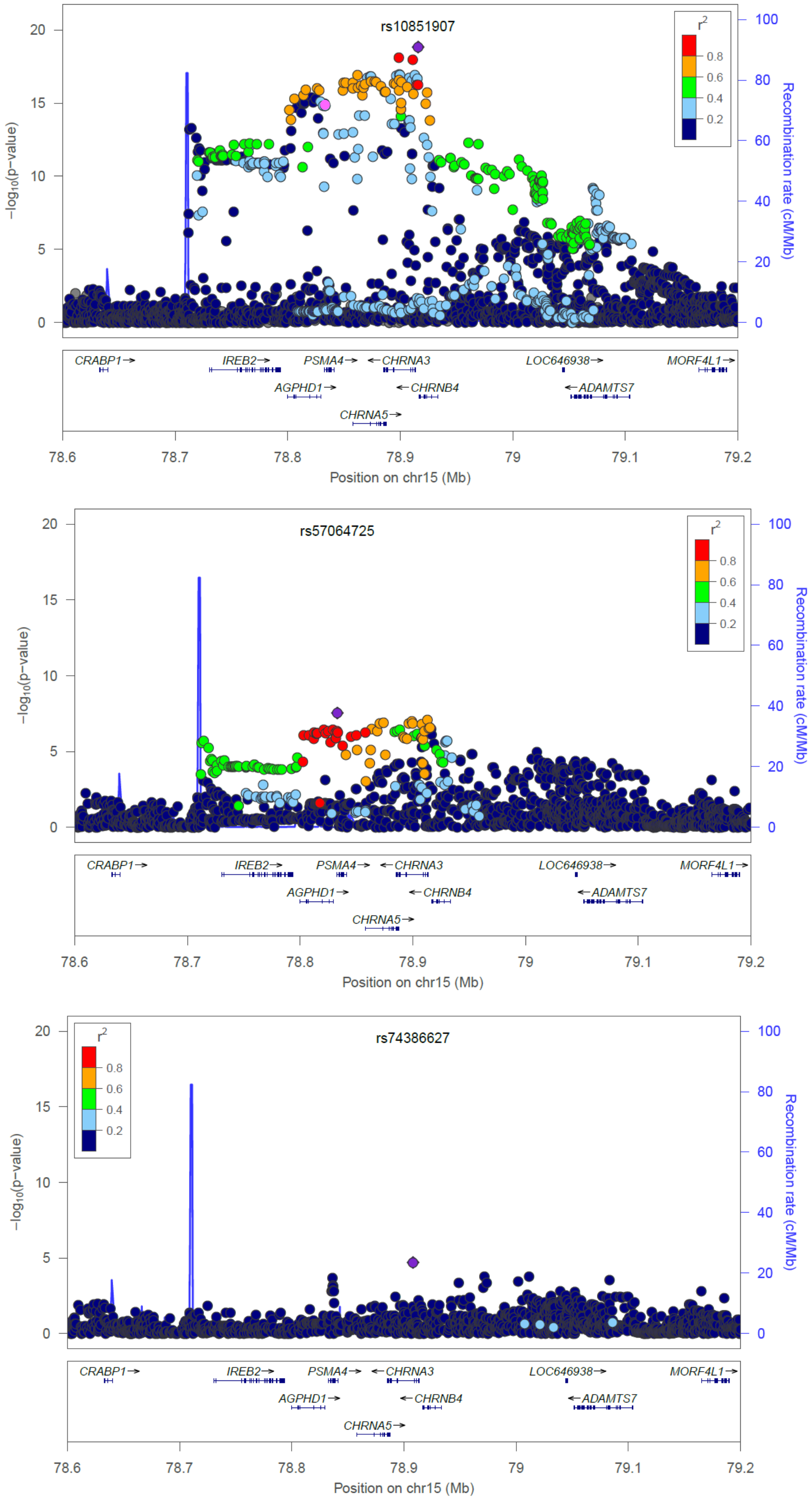

Figure S2. Chromosome 15 conditional analyses. Panels illustrate regional association plots of 15q25 region based on the original results (top), after conditioning on top hit rs10851907 (middle), and after conditioning on both rs10851907 and second independent signal rs57064725 (bottom). SNP rs57064725 is highlighted in pink in the top panel for reference. SNPs plotted by their position

on chromosome 15 against $–\log_{10}$ *p*-value for their association with cotinine level in genome-wide meta-analysis. The ALSPAC mothers cohort (n=8,890) was used as an LD reference panel for these analyses.

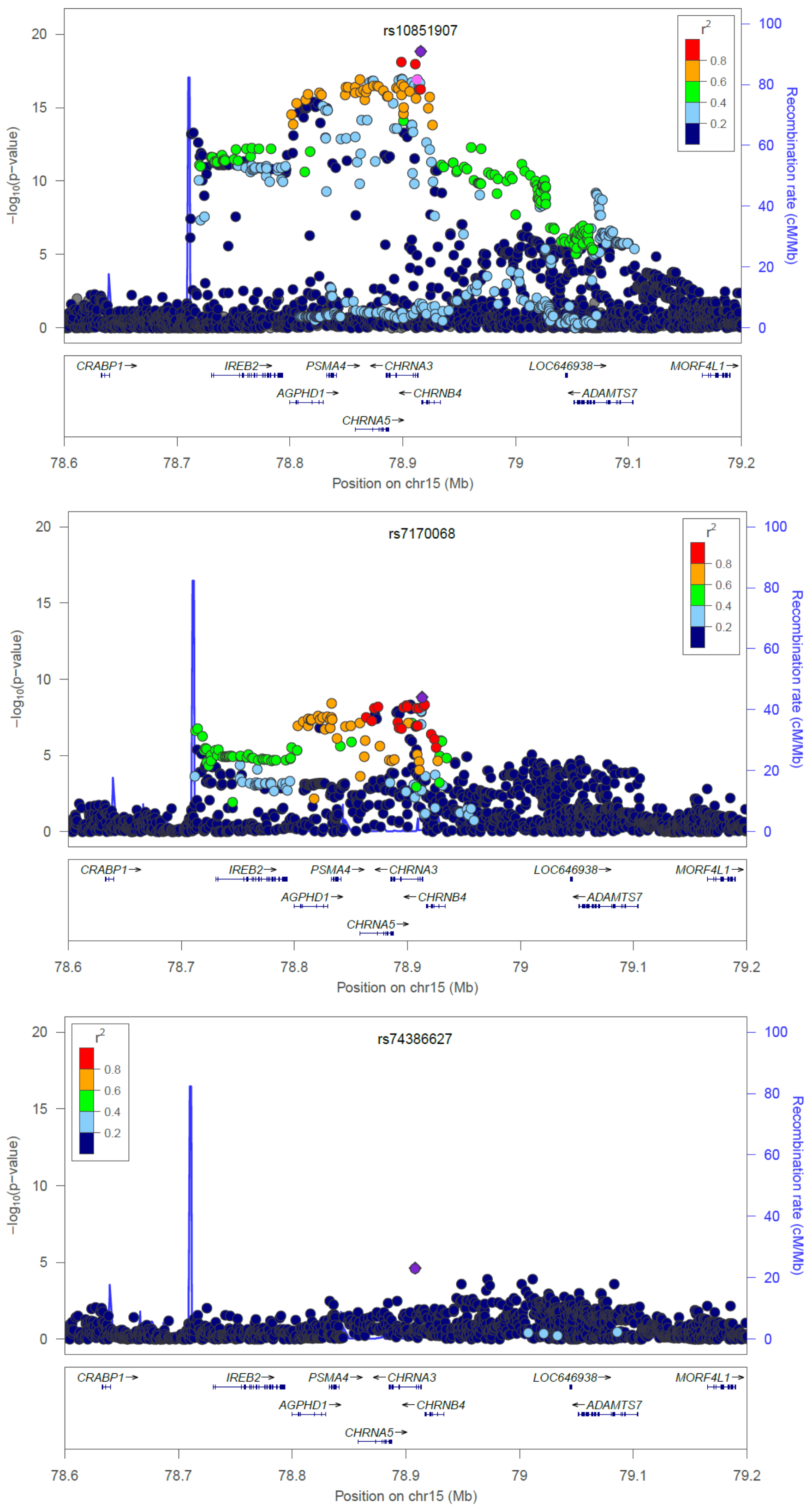

Figure S3. Chromosome 15 conditional analyses using rs16969968, which codes for D398N. Panels illustrate regional association plots of 15q25 region based on the original results (top), after conditioning on rs16969968 (middle), and after conditioning on both rs16969968 and second independent signal rs7170068 (bottom). SNP rs7170068 is highlighted in pink in the top panel for reference. SNPs plotted by their position on chromosome 15 against $-\log_{10}$ *p*-value for their association with cotinine level in genome-wide meta-analysis. The ALSPAC mothers cohort (n=8,890) was used as an LD reference panel for these analyses.

Table S1. Genotyping, imputation and statistical analysis for contributing studies.

Study	Genotyping					Imputation			Association analyses	
	Platform	Inclusion criteria			SNPs met QC criteria	Imputation software	Inclusion criteria			
		MAF	Call rate	P HWE			MAF	Imputation quality	SNPs in meta-analysis	λGC
ALSPAC	Illumina Human Hap 550-quad	≥1%	≥95%	>5x10-7	500,527	Minimac	>1%	$R^2 > 0.3$	7,999,998	0.993
CARDIA	Affy 6.0	>1%	>99%	>5x10-6	720,483	IMPUTE 2	>1%	Info > 0.4	8,227,647	1.005
FinnTwin[a]	Illumina 670K	>1%	>95%	>1x10-6	504,770+[a]	IMPUTE 2.2.2	>1%	Info > 0.4	8,585,905	1.006
FINRISK	Illumina HumanOmniExpress	>1%	>95%	>1x10-6	645,088	IMPUTE	>1%	Info > 0.4	6,030,573	1.025
Framingham	Affy 5.0	>1%	>99%	>5x10-6	500,571	IMPUTE 2	>1%	Info > 0.4	8,192,669	1.027
GenMets	Illumina 610K	>1%	>95%	>1x10-6	555,388	IMPUTE	>1%	Info > 0.4	8,520,923	1.011
MESA	Affy 6.0	>1%	>99%	>5x10-6	579,750	IMPUTE 2	>1%	Info > 0.4	8,466,568	1.009
NESDA	Perlegen-Affymetrix 5.0; Affymetrix 6.0 907K	>1%	≥90%	>1x10-5	250,000–1.1M	MACH 1.0	>1%	Info > 0.4	7,391,758	1.022
NTR	Perlegen-Affymetrix 5.0; Illumina 660K; Illumina 370K; Affymetrix 6.0 907K; Illumina Omni 1M	>1%	≥90%	>1x10-5	250,000-1.1M	MACH 1.0	>1%	Info > 0.4	5,253,422	0.995
TwinsUK	Illumina 317K+610K	≥1%	≥95%	>5x10-6	281,269	IMPUTE 2.2.0	>1%	Info > 0.4	8,392,704	1.012
YFS	Illumina 670K	≥1%	≥95%	>1x10-6	546,677	IMPUTE 2.1.2	>1%	Info > 0.4	8,583,652	0.997

[a]FinnTwin comprised three sub-samples, which contributed 549,060, 549,544, and 504,770 directly genotyped variants respectively.
MAF: minor allele frequency; QC: quality control; GC: genomic control.

Table S2. Summary information for all SNPs reaching or exceeding level of genome-wide significance.

SNP	Chr:Position	Gene	A1	A2	Freq A1	Beta	SE	p value	n
rs10851907	15:78915864	Intergenic (CHRNA3/CHRNB4)	a	g	0.4062	0.1862	0.0206	1.46E-19	4330
rs12914385	15:78898723	CHRNA3	t	c	0.3807	0.1837	0.0207	7.12E-19	4330
rs8040868	15:78911181	CHRNA3	t	c	0.6123	-0.1819	0.0206	9.96E-19	4330
rs7183604	15:78899213	CHRNA3	t	c	0.2419	-0.2058	0.024	1.02E-17	4330
rs7170068	15:78912943	CHRNA3	a	g	0.2239	-0.2142	0.025	1.13E-17	4330
rs11633958	15:78862064	CHRNA5	t	c	0.3307	0.2218	0.0259	1.15E-17	3433
rs11637630	15:78899719	CHRNA3	a	g	0.7591	0.205	0.024	1.20E-17	4330
rs938682	15:78896547	CHRNA3	a	g	0.759	0.2048	0.024	1.24E-17	4330
rs569207	15:78873119	CHRNA5	a	g	0.2409	-0.2043	0.0239	1.38E-17	4330
rs637137	15:78873976	CHRNA5	a	t	0.2409	-0.2043	0.0239	1.41E-17	4330
rs576982	15:78870803	CHRNA5	t	c	0.2381	-0.2065	0.0243	1.73E-17	4330
rs8042494	15:78908010	CHRNA3	t	c	0.2397	-0.2044	0.024	1.74E-17	4330
rs6495309	15:78915245	CHRNA3	t	c	0.2293	-0.2069	0.0244	1.94E-17	4330
rs55676755	15:78898932	CHRNA3	c	g	0.6649	-0.1815	0.0214	2.51E-17	4330
rs13329271	15:78914230	CHRNA3	a	c	0.7695	0.2064	0.0244	2.71E-17	4330
rs3825845	15:78910258	CHRNA3	t	c	0.2334	-0.2055	0.0243	2.82E-17	4330
rs56390833	15:78877381	CHRNA5	a	c	0.3331	0.1809	0.0214	3.04E-17	4330
rs7180002	15:78873993	CHRNA5	a	t	0.6656	-0.1803	0.0213	3.05E-17	4330
rs114205691	15:78901113	CHRNA3	t	c	0.3368	0.181	0.0215	3.42E-17	4330
rs951266	15:78878541	CHRNA5	t	c	0.3346	0.1799	0.0213	3.44E-17	4330
rs55853698	15:78857939	CHRNA5	t	g	0.6596	-0.183	0.0217	3.83E-17	4330
rs72740955	15:78849779	Intergenic (PSMA4/CHRNA5)	t	c	0.3378	0.1825	0.0217	4.00E-17	4330
rs2036527	15:78851615	Intergenic (PSMA4/CHRNA5)	a	g	0.3382	0.1823	0.0217	4.01E-17	4330
rs1051730	15:78894339	CHRNA3	a	g	0.3345	0.18	0.0214	4.15E-17	4330
rs1317286	15:78896129	CHRNA3	a	g	0.6638	-0.1801	0.0215	4.69E-17	4330
rs72740964	15:78868636	CHRNA5	a	g	0.3349	0.1798	0.0214	4.79E-17	4330
rs55958997	15:78915872	Intergenic (CHRNA3/CHRNB4)	a	c	0.3626	0.1784	0.0213	5.10E-17	4330
rs8039449	15:78914534	CHRNA3	t	c	0.3634	0.1778	0.0212	5.30E-17	4330
rs17486195	15:78865197	CHRNA5	a	g	0.6624	-0.1805	0.0216	5.68E-17	4330
rs16969968	15:78882925	CHRNA5	a	g	0.3343	0.1787	0.0214	6.91E-17	4330
rs146009840	15:78906177	CHRNA3	a	t	0.6659	-0.1796	0.0215	7.39E-17	4330
rs17486278	15:78867482	CHRNA5	a	c	0.6546	-0.1802	0.0216	8.01E-17	4330
rs7172118	15:78862453	CHRNA5	a	c	0.3371	0.18	0.0216	8.36E-17	4330
rs931794	15:78826180	AGPHD1	a	g	0.6581	-0.1772	0.0213	8.89E-17	4330
rs55781567	15:78857986	CHRNA5	c	g	0.6573	-0.1811	0.0218	9.16E-17	4330
rs8031948	15:78816057	AGPHD1	t	g	0.3395	0.1767	0.0213	1.16E-16	4330
rs56077333	15:78899003	CHRNA3	a	c	0.3177	0.1854	0.0224	1.29E-16	4330
rs72738786	15:78828086	AGPHD1	t	g	0.3387	0.178	0.0215	1.30E-16	4330
rs58365910	15:78849034	Intergenic (PSMA4/CHRNA5)	t	c	0.6579	-0.1798	0.0217	1.36E-16	4330
rs4887067	15:78886947	CHRNA3/CHRNA5	a	g	0.3349	0.1769	0.0214	1.56E-16	4330
rs8192482	15:78886198	CHRNA3/CHRNA5	t	c	0.3348	0.1768	0.0214	1.58E-16	4330
rs17487223	15:78923987	CHRNB4	a	g	0.3552	0.1747	0.0212	1.73E-16	4330
rs4243084	15:78911672	CHRNA3	c	g	0.3418	0.176	0.0214	2.25E-16	4330
rs140330585	15:78866445	CHRNA5	a	g	0.3391	0.1758	0.0215	2.90E-16	4330
rs10519203	15:78814046	AGPHD1	t	c	0.6597	-0.1742	0.0213	2.90E-16	4330
rs12441354	15:78821016	AGPHD1	a	g	0.2314	-0.1984	0.0244	4.01E-16	4330

rs8034191	15:78806023	AGPHD1	t	c	0.6584	-0.1725	0.0212	4.66E-16	4330
rs12438659	15:78824924	AGPHD1	a	g	0.2316	-0.1977	0.0244	4.87E-16	4330
rs12910984	15:78891627	CHRNA3	a	g	0.7582	0.1993	0.0246	5.02E-16	4072
rs12439240	15:78829091	AGPHD1	t	c	0.7782	0.2103	0.0261	7.28E-16	4330
rs7169584	15:78822660	AGPHD1	a	t	0.7686	0.196	0.0244	8.24E-16	4330
rs7163730	15:78814681	AGPHD1	a	g	0.7686	0.1961	0.0244	8.58E-16	4330
rs61204066	15:78815298	AGPHD1	a	g	0.2315	-0.196	0.0244	8.64E-16	4330
rs12441426	15:78812329	AGPHD1	t	c	0.7685	0.1962	0.0244	9.05E-16	4330
rs147144681	15:78900908	CHRNA3	t	c	0.3088	0.1847	0.023	9.42E-16	4330
rs7181245	15:78814389	AGPHD1	t	c	0.2316	-0.1957	0.0244	9.60E-16	4330
rs7181447	15:78814567	AGPHD1	a	g	0.7683	0.1956	0.0244	9.93E-16	4330
rs3813570	15:78832832	PSMA4	t	c	0.777	0.2087	0.026	1.02E-15	4330
rs2869548	15:78922638	CHRNB4	a	g	0.3584	0.2055	0.0256	1.09E-15	3433
rs57064725	15:78833036	PSMA4	a	c	0.248	-0.2422	0.0303	1.33E-15	3433
rs59683676	15:78833453	PSMA4	t	c	0.7769	0.2079	0.0261	1.51E-15	4330
rs59133824	15:78833450	PSMA4	a	c	0.223	-0.2081	0.0261	1.51E-15	4330
rs34664138	15:78831624	PSMA4	t	g	0.7799	0.2085	0.0261	1.53E-15	4330
rs28437878	15:78807872	AGPHD1	t	c	0.232	-0.1939	0.0244	1.95E-15	4330
rs141518190	15:78900647	CHRNA3	a	g	0.6998	-0.1859	0.0235	2.59E-15	4330
rs147499554	15:78900650	CHRNA3	t	c	0.3002	0.1859	0.0235	2.59E-15	4330
rs11852372	15:78801394	AGPHD1	a	c	0.6813	-0.1768	0.0224	2.95E-15	4330
rs12438181	15:78812098	AGPHD1	a	g	0.2268	-0.196	0.025	4.50E-15	4330
rs667282	15:78863472	CHRNA5	t	c	0.7582	0.2378	0.0305	6.45E-15	3433
rs2456020	15:78868398	CHRNA5	t	c	0.2439	-0.2268	0.0291	6.81E-15	3433
rs138544659	15:78900701	CHRNA3	t	g	0.7072	-0.1867	0.024	7.23E-15	4330
rs2036534	15:78826948	AGPHD1	t	c	0.7669	0.1919	0.0248	1.09E-14	4072
rs9788721	15:78802869	AGPHD1	t	c	0.6535	-0.1638	0.0213	1.27E-14	4330
rs72743158	15:78926445	CHRNB4	t	c	0.6316	-0.1982	0.0258	1.45E-14	3433
rs8042374	15:78908032	CHRNA3	a	g	0.7549	0.2192	0.0285	1.51E-14	3433
rs6495308	15:78907656	CHRNA3	a	g	0.7546	0.2187	0.0285	1.53E-14	3433
rs8043009	15:78908154	CHRNA3	c	g	0.2453	-0.219	0.0285	1.53E-14	3433
rs8042059	15:78907859	CHRNA3	a	c	0.7547	0.2187	0.0285	1.55E-14	3433
rs7177514	15:78907406	CHRNA3	c	g	0.7549	0.2184	0.0285	1.79E-14	3433
rs3743078	15:78894759	CHRNA3	c	g	0.2478	-0.2165	0.0284	2.60E-14	3433
rs7359276	15:78892661	CHRNA3	t	c	0.751	0.217	0.0285	2.65E-14	3433
rs4887069	15:78909070	CHRNA3	a	g	0.7478	0.2162	0.0286	4.17E-14	3433
rs2869032	15:78714561	Intergenic (IREB2)	t	c	0.791	0.191	0.0253	4.81E-14	4330
rs7171869	15:78900909	CHRNA3	a	g	0.2815	-0.1848	0.0245	4.94E-14	4330
rs4887053	15:78712699	Intergenic (IREB2)	a	c	0.2092	-0.1887	0.0251	6.12E-14	4330
rs7164594	15:78803057	AGPHD1	t	c	0.2379	-0.2165	0.0289	7.26E-14	3433
rs189218934	15:78903987	CHRNA3	t	c	0.3801	-0.1911	0.0256	9.42E-14	4330
rs503464	15:78857896	CHRNA5	a	t	0.2642	-0.23	0.0309	9.76E-14	3433
rs34138960	15:78831668	PSMA4	a	g	0.7685	0.2222	0.0299	1.05E-13	3433
rs35212593	15:78831826	PSMA4	a	g	0.2314	-0.2222	0.0299	1.12E-13	3433
rs7173514	15:78849918	Intergenic (PSMA4/CHRNA5)	t	c	0.2438	-0.2324	0.0313	1.13E-13	3433
rs905739	15:78845110	Intergenic (PSMA4/CHRNA5)	a	g	0.766	0.2323	0.0314	1.32E-13	3433
rs2869045	15:78718899	Intergenic (IREB2)	t	c	0.2083	-0.187	0.0255	2.15E-13	4330

rs12593950	15:78920935	CHRNB4	c	g	0.2409	-0.2188	0.0298	2.30E-13	3433
rs6495314	15:78960529	Intergenic (CHRNB4)	a	c	0.5956	-0.15	0.0207	4.76E-13	4330
rs1504550	15:78766250	IREB2	a	g	0.6736	-0.1556	0.0216	5.46E-13	4330
rs17484524	15:78772676	IREB2	a	g	0.673	-0.1553	0.0216	5.87E-13	4330
rs17484235	15:78761414	IREB2	c	g	0.6733	-0.155	0.0215	5.94E-13	4330
rs11858836	15:78783277	IREB2	a	g	0.3234	0.1564	0.0217	6.23E-13	4330
rs4886580	15:78969385	Intergenic (CHRNB4)	t	g	0.6002	-0.1499	0.0208	6.36E-13	4330
rs28534575	15:78923845	CHRNB4	t	g	0.759	0.2156	0.03	6.45E-13	3433
rs72738732	15:78752188	IREB2	c	g	0.6739	-0.1547	0.0215	6.50E-13	4330
rs12592111	15:78767346	IREB2	a	g	0.5911	0.1537	0.0214	6.66E-13	4330
rs8042849	15:78817929	AGPHD1	t	c	0.5962	-0.1589	0.0223	9.09E-13	4330
rs2568497	15:78721397	Intergenic (IREB2)	t	g	0.7818	0.1776	0.025	1.18E-12	4330
rs9788682	15:78802586	AGPHD1	a	g	0.2147	-0.1771	0.0249	1.21E-12	4330
rs664172	15:78862762	CHRNA5	a	g	0.2066	-0.2432	0.0343	1.34E-12	3433
rs9672608	15:78797463	Intergenic (IREB2/AGPHD1)	a	t	0.231	-0.1753	0.0248	1.57E-12	4330
rs2568499	15:78722359	Intergenic (IREB2)	t	c	0.2195	-0.176	0.0249	1.61E-12	4330
rs2089162	15:78739763	IREB2	a	g	0.6726	-0.1527	0.0216	1.66E-12	4330
rs518425	15:78883813	CHRNA5	a	g	0.7041	0.1912	0.0271	1.67E-12	3433
rs72738736	15:78765122	IREB2	t	g	0.332	0.1511	0.0215	1.98E-12	4330
rs578776	15:78888400	CHRNA3	a	g	0.2908	-0.1915	0.0272	2.05E-12	3433
rs2656055	15:78720194	Intergenic (IREB2)	a	c	0.7832	0.1766	0.0251	2.14E-12	4330
rs17405217	15:78731149	IREB2	t	c	0.3262	0.1524	0.0217	2.17E-12	4237
rs17483548	15:78730313	IREB2	a	g	0.3262	0.1523	0.0217	2.18E-12	4237
rs2656070	15:78730252	IREB2	a	g	0.2149	-0.179	0.0255	2.26E-12	4330
rs2938671	15:78732754	IREB2	a	g	0.2388	-0.1709	0.0244	2.61E-12	4330
rs8192477	15:78910463	CHRNA3	c	g	0.2588	-0.1719	0.0246	2.73E-12	4330
rs8192478	15:78910462	CHRNA3	a	g	0.7415	0.1719	0.0246	2.80E-12	4330
rs564585	15:78886227	CHRNA3/CHRNA5	a	g	0.7109	0.1913	0.0274	2.90E-12	3433
rs2656065	15:78750549	IREB2	a	g	0.3375	0.1502	0.0215	3.16E-12	4330
rs17483929	15:78742376	IREB2	t	c	0.3309	0.1493	0.0214	3.22E-12	4330
rs56219465	15:78742579	IREB2	a	g	0.6691	-0.1489	0.0214	3.76E-12	4330
rs2009746	15:78754102	IREB2	a	g	0.6637	-0.1501	0.0216	3.78E-12	4330
rs55983731	15:78735269	IREB2	t	c	0.3308	0.1488	0.0214	3.91E-12	4330
rs2938670	15:78740688	IREB2	t	g	0.6682	-0.1488	0.0214	3.93E-12	4330
rs2656052	15:78740964	IREB2	a	c	0.6682	-0.1488	0.0214	3.93E-12	4330
rs2568494	15:78740932	IREB2	a	g	0.3318	0.1488	0.0214	3.93E-12	4330
rs11637656	15:78751961	IREB2	t	c	0.6047	0.1458	0.021	3.94E-12	4330
rs28395178	15:78850558	Intergenic (PSMA4/CHRNA5)	a	g	0.2	-0.2344	0.0338	4.07E-12	3433
rs7181486	15:78741618	IREB2	t	c	0.6692	-0.1486	0.0214	4.11E-12	4330
rs1700006	15:78875623	CHRNA5	a	g	0.8093	0.2403	0.0347	4.46E-12	3433
rs17483721	15:78733731	IREB2	t	c	0.6692	-0.1484	0.0215	4.54E-12	4330
rs958025	15:78759348	IREB2	t	c	0.2172	-0.1741	0.0252	4.57E-12	4330
rs4887072	15:78925435	CHRNB4	a	g	0.7659	0.2138	0.0309	4.70E-12	3433
rs72738718	15:78735438	IREB2	c	g	0.33	0.1485	0.0215	4.86E-12	4330
rs17483686	15:78733390	IREB2	a	t	0.6694	-0.1481	0.0215	5.06E-12	4330
rs190065944	15:78859610	CHRNA5	a	g	0.2745	0.2316	0.0336	5.12E-12	3433
rs11634351	15:78944718	Intergenic (RPL18P11/CHRNB4)	a	g	0.3973	0.1759	0.0255	5.22E-12	3433

rs924840	15:78731808	IREB2	a	t	0.7747	0.1681	0.0244	5.39E-12	4330
rs11633178	15:78944538	Intergenic (CHRNB4)	a	g	0.6018	-0.1759	0.0255	5.46E-12	3433
rs2568483	15:78752343	IREB2	a	g	0.7762	0.1679	0.0244	6.43E-12	4330
rs11634543	15:79005504	Intergenic (LOC646934/ADAMTS7)	t	c	0.4001	0.1831	0.0267	6.58E-12	3433
rs2958719	15:78743029	IREB2	a	g	0.7762	0.1677	0.0244	6.62E-12	4330
rs2915695	15:78739471	IREB2	t	c	0.2237	-0.1676	0.0244	6.77E-12	4330
rs2656072	15:78744292	IREB2	a	g	0.221	-0.1702	0.0248	7.08E-12	4330
rs2656069	15:78745707	IREB2	t	c	0.7762	0.1674	0.0244	7.20E-12	4330
rs2568493	15:78740233	IREB2	a	g	0.7762	0.1673	0.0244	7.38E-12	4330
rs67426328	15:78934318	CHRNB4	c	g	0.6041	-0.1781	0.026	7.42E-12	3433
rs2656074	15:78741384	IREB2	t	c	0.2237	-0.1673	0.0244	7.46E-12	4330
rs72738704	15:78719832	Intergenic (IREB2)	c	g	0.3275	0.1514	0.0221	7.55E-12	4330
rs2656073	15:78742276	IREB2	t	g	0.2237	-0.1672	0.0244	7.57E-12	4330
rs2568485	15:78752114	IREB2	t	c	0.7797	0.171	0.025	7.60E-12	4330
rs2568488	15:78736593	IREB2	a	t	0.7764	0.1673	0.0244	7.63E-12	4330
rs2568490	15:78738370	IREB2	t	c	0.2237	-0.1672	0.0244	7.63E-12	4330
rs1964678	15:78754000	IREB2	a	g	0.3883	-0.1447	0.0212	8.16E-12	4330
rs28480606	15:78762313	IREB2	a	g	0.7795	0.1697	0.0249	8.75E-12	4330
rs4299116	15:78766194	IREB2	a	t	0.6075	0.143	0.021	8.80E-12	4330
rs2938674	15:78757913	IREB2	a	c	0.221	-0.1699	0.0249	8.80E-12	4330
rs951985	15:78720923	Intergenic (IREB2)	t	g	0.692	-0.1607	0.0236	9.12E-12	4330
rs12904234	15:78779384	IREB2	t	c	0.6125	0.1433	0.021	9.31E-12	4330
rs4362358	15:78796104	Intergenic (IREB2/AGPHD1)	t	c	0.6109	0.1454	0.0213	9.33E-12	4330
rs7174190	15:78763617	IREB2	t	c	0.2184	-0.1705	0.025	9.38E-12	4330
rs12903295	15:78778972	IREB2	a	g	0.3897	-0.1428	0.021	9.46E-12	4330
rs77438389	15:78726271	Intergenic (IREB2)	c	g	0.766	0.1687	0.0248	1.03E-11	4330
rs12899135	15:78954379	Intergenic (RPL18P11/CHRNB4)	a	g	0.6076	-0.1718	0.0253	1.05E-11	3433
rs55988292	15:78936168	Intergenic (CHRNB4)	a	g	0.5987	-0.1763	0.0259	1.06E-11	3433
rs12899351	15:78792398	IREB2	t	c	0.3919	-0.1418	0.0209	1.14E-11	4330
rs8042260	15:78774374	IREB2	a	g	0.3913	-0.142	0.0209	1.17E-11	4330
rs11072766	15:78771546	IREB2	t	c	0.2241	-0.1658	0.0244	1.18E-11	4330
rs8042238	15:78774271	IREB2	t	c	0.6087	0.1419	0.0209	1.18E-11	4330
rs13180	15:78789488	IREB2	t	c	0.608	0.1417	0.0209	1.18E-11	4330
rs113352275	15:78840567	PSMA4	t	c	0.2005	-0.2268	0.0334	1.18E-11	3433
rs12916801	15:78769130	IREB2	a	g	0.3891	-0.1425	0.021	1.19E-11	4330
rs35031105	15:78760918	IREB2	t	c	0.2242	-0.1656	0.0244	1.24E-11	4330
rs4887057	15:78772806	IREB2	a	g	0.3869	-0.1432	0.0211	1.24E-11	4330
rs36146269	15:78779510	IREB2	a	t	0.6089	0.1417	0.0209	1.26E-11	4330
rs10851906	15:78774676	IREB2	a	g	0.7759	0.1654	0.0244	1.28E-11	4330
rs16969894	15:78776456	IREB2	t	c	0.2245	-0.1656	0.0245	1.29E-11	4330
rs2036533	15:78781687	IREB2	a	g	0.2241	-0.165	0.0244	1.41E-11	4330
rs12441998	15:78929372	CHRNB4	a	g	0.7754	0.2117	0.0313	1.45E-11	3433
rs28602670	15:78768167	IREB2	c	g	0.7814	0.1689	0.025	1.46E-11	4330
rs28511883	15:78783683	IREB2	t	c	0.2244	-0.1646	0.0244	1.53E-11	4330
rs8043227	15:78768871	IREB2	c	g	0.3893	-0.1415	0.021	1.54E-11	4330
rs1996371	15:78956806	Intergenic (RPL18P11/CHRNB4)	t	c	0.609	-0.1695	0.0251	1.54E-11	3433
rs1504549	15:78766629	IREB2	t	c	0.6105	0.1413	0.021	1.56E-11	4330

rs12101809	15:78779801	IREB2	t	c	0.2241	-0.1645	0.0244	1.61E-11	4330
rs7174348	15:78792439	IREB2	a	g	0.2118	-0.172	0.0255	1.64E-11	4330
rs12910910	15:78767850	IREB2	t	c	0.6108	0.1413	0.021	1.64E-11	4330
rs74925218	15:78796732	Intergenic (IREB2/AGPHD1)	t	c	0.2329	-0.1671	0.0248	1.68E-11	4330
rs11638830	15:78948319	Intergenic (RPL18P11/CHRNB4)	c	g	0.3957	0.1703	0.0253	1.74E-11	3433
rs965604	15:78789223	IREB2	a	g	0.6098	0.1408	0.0209	1.80E-11	4330
rs1316971	15:78930510	CHRNB4	a	g	0.2241	-0.2111	0.0314	1.80E-11	3433
rs11072768	15:78929478	CHRNB4	a	c	0.224	-0.2107	0.0314	1.81E-11	3433
rs11636605	15:78928878	CHRNB4	a	g	0.2243	-0.2105	0.0314	1.93E-11	3433
rs11638490	15:79007950	Intergenic (LOC646934/ADAMTS7)	t	c	0.3938	0.1791	0.0267	2.04E-11	3433
rs12593229	15:78765290	IREB2	t	g	0.3857	-0.1417	0.0212	2.18E-11	4330
rs2170311	15:78947611	Intergenic (RPL18P11/CHRNB4)	a	g	0.3952	0.1703	0.0255	2.25E-11	3433
rs34684276	15:78813155	AGPHD1	a	g	0.2725	0.1974	0.0295	2.32E-11	3433
rs5019044	15:78796282	Intergenic (IREB2/AGPHD1)	a	t	0.2065	-0.1768	0.0265	2.44E-11	4330
rs72743199	15:78975855	Intergenic (RPL18P11/CHRNB4)	a	t	0.6182	-0.1706	0.0256	2.51E-11	3433
rs28681284	15:78908565	CHRNA3	t	c	0.1858	-0.2362	0.0355	2.92E-11	3433
rs2036529	15:78726272	Intergenic (IREB2)	a	t	0.2306	-0.1644	0.0248	3.12E-11	4330
rs12901913	15:78981348	Intergenic (LOC646934/ADAMTS7/CHRNB4)	t	c	0.3737	0.1725	0.0261	3.62E-11	3433
rs4887077	15:78978364	Intergenic (LOC646934/ADAMTS7/CHRNB4)	a	g	0.3803	0.1684	0.0254	3.68E-11	3433
rs113134286	15:79012888	Intergenic (LOC646934/ADAMTS7)	t	c	0.3862	0.176	0.0266	3.92E-11	3433
rs12910627	15:78994933	Intergenic (LOC646934/ADAMTS7)	c	g	0.3845	0.1783	0.0271	4.34E-11	3433
rs12903285	15:78778953	IREB2	a	g	0.4207	-0.1425	0.0216	4.59E-11	4330
rs11072791	15:78997076	Intergenic (LOC646934/ADAMTS7)	a	c	0.3742	0.1773	0.0272	6.96E-11	3433
rs922692	15:78984214	Intergenic (LOC646934/ADAMTS7/CHRNB4)	a	c	0.3728	0.1704	0.0261	7.16E-11	3433
rs1062980	15:78792527	IREB2	t	c	0.6092	0.14	0.0215	7.40E-11	4072
rs4887083	15:79025577	LOC646934	t	c	0.3798	0.1902	0.0293	8.30E-11	3433
rs4420500	15:78962964	Intergenic (RPL18P11/CHRNB4)	t	c	0.3851	0.1685	0.0259	8.31E-11	3433
rs2656056	15:78722519	IREB2	t	c	0.2438	-0.1624	0.025	8.64E-11	4330
rs72736802	15:78719501	Intergenic (IREB2)	a	t	0.6554	-0.1521	0.0235	8.82E-11	4330
rs11072790	15:78992025	Intergenic (LOC646934/ADAMTS7/CHRNB4)	t	c	0.3868	0.1742	0.0269	9.23E-11	3433
rs8038543	15:79017685	Intergenic (LOC646934/ADAMTS7)	a	t	0.616	-0.1742	0.0269	9.34E-11	3433
rs2869561	15:79025516	LOC646934	t	c	0.6212	-0.1897	0.0293	9.98E-11	3433
rs12440014	15:78926726	CHRNB4	c	g	0.7622	0.1987	0.0307	1.03E-10	3433
rs12594711	15:78793921	IREB2	t	c	0.6036	0.1367	0.0211	1.04E-10	4330
rs4887059	15:78782095	IREB2	t	c	0.6112	0.1363	0.0211	1.04E-10	4330
rs11639372	15:78966655	Intergenic (RPL18P11/CHRNB4)	t	c	0.386	0.1605	0.025	1.27E-10	3433
rs12902602	15:78967401	Intergenic (RPL18P11/CHRNB4)	a	g	0.6151	-0.1603	0.0249	1.31E-10	3433
rs1021071	15:78968179	Intergenic (RPL18P11/CHRNB4)	c	g	0.3851	0.1602	0.0249	1.31E-10	3433
rs76919723	15:79017281	Intergenic (LOC646934/ADAMTS7)	t	c	0.6211	-0.1738	0.0271	1.35E-10	3433
rs4886579	15:78969256	Intergenic (RPL18P11/CHRNB4)	t	c	0.3851	0.1598	0.0249	1.48E-10	3433
rs11072785	15:78968229	Intergenic (RPL18P11/CHRNB4)	t	c	0.385	0.1597	0.0249	1.54E-10	3433
rs28669908	15:78910267	CHRNA3	a	c	0.1779	-0.2313	0.0361	1.54E-10	3433
rs11629637	15:79019024	Intergenic (LOC646934/ADAMTS7)	t	c	0.3837	0.1708	0.0268	1.86E-10	3433
rs11639375	15:79024214	LOC646934	a	g	0.3774	0.1777	0.0279	1.89E-10	3433
rs11639382	15:79024268	LOC646934	a	g	0.3774	0.1778	0.0279	1.89E-10	3433
rs11639347	15:79024350	LOC646934	t	c	0.3774	0.1778	0.0279	1.90E-10	3433
rs1825082	15:79023433	LOC646934	a	g	0.3781	0.1767	0.0277	1.90E-10	3433

rs11639181	15:79024016	LOC646934	a	g	0.6226	-0.1777	0.0279	1.90E-10	3433
rs11639049	15:79023730	LOC646934	a	g	0.6222	-0.1769	0.0278	1.94E-10	3433
rs1825083	15:79023467	LOC646934	t	c	0.6221	-0.1768	0.0278	1.97E-10	3433
rs11639166	15:79023766	LOC646934	c	g	0.3777	0.1768	0.0278	1.97E-10	3433
rs12908207	15:79025963	LOC646934	t	g	0.3716	0.1873	0.0295	2.22E-10	3433
rs4886587	15:79026822	LOC646934	a	t	0.3774	0.1871	0.0295	2.33E-10	3433
rs3894347	15:79026001	LOC646934	a	c	0.3635	0.1907	0.0301	2.38E-10	3433
rs4887086	15:79026529	LOC646934	t	c	0.3782	0.1857	0.0294	2.77E-10	3433
rs3829786	15:79019546	Intergenic (LOC646934/ADAMTS7)	a	c	0.3818	0.1672	0.0267	3.56E-10	3433
rs56117933	15:78832349	PSMA4	t	c	0.8074	-0.21	0.0338	4.92E-10	3433
rs34225855	15:79022136	LOC646934	c	g	0.6231	-0.1721	0.0277	4.99E-10	3433
rs67175876	15:78928399	CHRNB4	a	g	0.7667	0.1963	0.0316	5.06E-10	3433
rs114612145	4:69746647	Intergenic (UGT2B10/UGT2A3)	a	g	0.9041	-0.2221	0.0359	5.89E-10	4290
rs116692755	4:69746875	Intergenic (UGT2B10/UGT2A3)	c	g	0.9041	-0.2221	0.0359	5.90E-10	4290
rs3813567	15:78934551	CHRNB4	a	g	0.7751	0.1978	0.032	5.95E-10	3433
rs12913260	15:79071095	ADAMTS7	a	g	0.359	0.1622	0.0262	6.16E-10	4330
rs116115181	4:69717641	Intergenic (UGT2B10/UGT2A3)	t	c	0.0883	0.2305	0.0373	6.29E-10	4290
rs11638372	15:78983559	Intergenic (LOC646934/ADAMTS7/CHRNB4)	a	g	0.3647	0.1618	0.0262	6.74E-10	3433
rs115319921	4:69721662	Intergenic (UGT2B10/UGT2A3)	t	c	0.9116	-0.2281	0.037	7.37E-10	4290
rs150405933	4:69735957	Intergenic (UGT2B10/UGT2A3)	t	c	0.0899	0.226	0.0367	7.40E-10	4290
rs2004038	15:79071406	ADAMTS7	a	g	0.3844	0.1601	0.026	7.42E-10	4330
rs115542835	4:69725113	Intergenic (UGT2B10/UGT2A3)	t	g	0.0884	0.2278	0.037	7.49E-10	4290
rs114473265	4:69739411	Intergenic (UGT2B10/UGT2A3)	t	c	0.9103	-0.2254	0.0367	7.89E-10	4290
rs115612883	4:69742722	Intergenic (UGT2B10/UGT2A3)	a	g	0.9103	-0.2255	0.0367	7.92E-10	4290
rs116018225	4:69740155	Intergenic (UGT2B10/UGT2A3)	t	c	0.0898	0.2253	0.0367	7.98E-10	4290
rs113997869	4:69735877	Intergenic (UGT2B10/UGT2A3)	t	c	0.089	0.2258	0.0368	8.06E-10	4290
rs35583595	15:79020152	Intergenic (LOC646934/ADAMTS7)	c	g	0.6192	-0.1627	0.0265	8.36E-10	3433
rs112321636	15:79072100	ADAMTS7	t	g	0.3797	0.16	0.0261	8.55E-10	4330
rs115293372	4:69745127	Intergenic (UGT2B10/UGT2A3)	t	c	0.91	-0.2245	0.0366	8.64E-10	4290
rs11857532	15:78968268	Intergenic (CHRNB4)	a	c	0.5546	-0.1256	0.0205	8.65E-10	4330
rs115970480	4:69752439	Intergenic (UGT2B10/UGT2A3)	t	c	0.0903	0.2231	0.0365	9.46E-10	4290
rs116221291	4:69750117	LOC100174950	t	c	0.9097	-0.223	0.0365	9.51E-10	4290
rs11072763	15:78724256	Intergenic (IREB2)	a	g	0.2424	-0.177	0.0289	9.56E-10	3433
rs115815311	4:69753685	Intergenic (UGT2B10/UGT2A3)	a	g	0.0911	0.2219	0.0363	9.77E-10	4290
rs115549616	4:69751951	Intergenic (UGT2B10/UGT2A3)	c	g	0.9096	-0.2227	0.0364	9.86E-10	4290
rs115102804	4:69710615	Intergenic (UGT2B10/UGT2A3)	t	g	0.0882	0.2283	0.0374	1.04E-09	4290
rs139784893	4:69749552	LOC100174950	a	g	0.0837	0.238	0.039	1.05E-09	4290
rs116379644	4:69755206	Intergenic (UGT2B10/UGT2A3)	a	c	0.9089	-0.2212	0.0363	1.06E-09	4290
rs115166258	4:69754283	Intergenic (UGT2B10/UGT2A3)	a	g	0.0908	0.2214	0.0363	1.12E-09	4290
rs116380956	4:69759101	Intergenic (UGT2B10/UGT2A3)	a	g	0.9091	-0.2211	0.0363	1.13E-09	4290
rs114014249	4:69760471	Intergenic (UGT2B10/UGT2A3)	a	g	0.9095	-0.2209	0.0364	1.24E-09	4290
rs113996790	4:69769087	Intergenic (UGT2B10/UGT2A3)	t	c	0.0921	0.2208	0.0363	1.24E-09	4290
rs116446865	4:69767349	Intergenic (UGT2B10/UGT2A3)	t	c	0.9091	-0.2208	0.0364	1.25E-09	4290
rs114441621	4:69761131	Intergenic (UGT2B10/UGT2A3)	t	c	0.9097	-0.2213	0.0364	1.26E-09	4290
rs115707640	4:69759351	Intergenic (UGT2B10/UGT2A3)	t	c	0.0904	0.221	0.0364	1.27E-09	4290
rs114126014	4:69768638	Intergenic (UGT2B10/UGT2A3)	t	c	0.9094	-0.2209	0.0364	1.31E-09	4290
rs116023959	4:69766993	Intergenic (UGT2B10/UGT2A3)	t	g	0.0907	0.2211	0.0364	1.31E-09	4290

rs115558925	4:69765782	Intergenic (UGT2B10/UGT2A3)	a	c	0.9095	-0.2209	0.0364	1.31E-09	4290
rs116192600	4:69769999	Intergenic (UGT2B10/UGT2A3)	c	g	0.9083	-0.2205	0.0364	1.35E-09	4290
rs4886591	15:79074518	ADAMTS7	a	g	0.3969	0.1542	0.0255	1.40E-09	4330
rs116144535	4:69722395	Intergenic (UGT2B10/UGT2A3)	a	g	0.0992	0.212	0.035	1.41E-09	4290
rs34563625	15:79020278	Intergenic (LOC646934/ADAMTS7)	c	g	0.3709	0.164	0.0271	1.51E-09	3433
rs3813565	15:79019610	Intergenic (LOC646934/ADAMTS7)	t	g	0.3768	0.1597	0.0264	1.52E-09	3433
rs188560849	4:69717968	Intergenic (UGT2B10/UGT2A3)	a	c	0.0835	0.2337	0.0388	1.66E-09	4290
rs192169379	4:69717969	Intergenic (UGT2B10/UGT2A3)	a	t	0.9165	-0.2337	0.0388	1.66E-09	4290
rs114373813	4:69724935	Intergenic (UGT2B10/UGT2A3)	a	g	0.9166	-0.2326	0.0387	1.78E-09	4290
rs2869554	15:79021037	Intergenic (LOC646934/ADAMTS7)	t	c	0.6362	-0.1695	0.0282	1.83E-09	3433
rs2869861	15:79076744	ADAMTS7	a	g	0.6455	-0.1485	0.0247	1.92E-09	4330
rs116285470	4:69716229	Intergenic (UGT2B10/UGT2A3)	a	c	0.0942	0.214	0.0357	1.97E-09	4290
rs115952417	4:69717836	Intergenic (UGT2B10/UGT2A3)	a	t	0.9061	-0.2138	0.0356	1.98E-09	4290
rs114858759	4:69727258	Intergenic (UGT2B10/UGT2A3)	c	g	0.0935	0.2132	0.0356	2.03E-09	4290
rs114711302	4:69727220	Intergenic (UGT2B10/UGT2A3)	t	c	0.0935	0.2132	0.0356	2.03E-09	4290
rs116372253	4:69728015	Intergenic (UGT2B10/UGT2A3)	a	t	0.9067	-0.2136	0.0356	2.04E-09	4290
rs115292836	4:69731361	Intergenic (UGT2B10/UGT2A3)	a	t	0.0937	0.2125	0.0354	2.05E-09	4290
rs116286898	4:69719579	Intergenic (UGT2B10/UGT2A3)	a	g	0.0938	0.2134	0.0356	2.06E-09	4290
rs115933485	4:69725968	Intergenic (UGT2B10/UGT2A3)	t	c	0.0933	0.2136	0.0357	2.07E-09	4290
rs115109494	4:69732112	Intergenic (UGT2B10/UGT2A3)	a	t	0.0934	0.213	0.0355	2.09E-09	4290
rs116118888	4:69731469	Intergenic (UGT2B10/UGT2A3)	c	g	0.0934	0.2129	0.0355	2.11E-09	4290
rs114866244	4:69723767	Intergenic (UGT2B10/UGT2A3)	t	c	0.9062	-0.2123	0.0355	2.12E-09	4290
rs116498746	4:69722960	Intergenic (UGT2B10/UGT2A3)	t	c	0.0938	0.2124	0.0355	2.12E-09	4290
rs116020958	4:69721871	Intergenic (UGT2B10/UGT2A3)	a	g	0.0935	0.213	0.0356	2.16E-09	4290
rs80229697	15:79022616	LOC646934	a	g	0.3461	0.1761	0.0295	2.22E-09	3433
rs116017934	4:69727682	Intergenic (UGT2B10/UGT2A3)	a	g	0.0941	0.2164	0.0362	2.32E-09	4072
rs115264860	4:69725006	Intergenic (UGT2B10/UGT2A3)	t	c	0.0941	0.2164	0.0362	2.33E-09	4072
rs8027972	15:79022615	LOC646934	t	c	0.6541	-0.1759	0.0295	2.37E-09	3433
rs4886582	15:79021441	LOC646934	a	g	0.6486	-0.1702	0.0286	2.67E-09	3433
rs116011053	4:69707693	Intergenic (UGT2B10/UGT2A3)	a	c	0.0824	0.2388	0.0402	2.95E-09	4290
rs138914938	15:79073454	ADAMTS7	c	g	0.3854	0.1498	0.0253	3.07E-09	4330
rs191592946	4:69803723	UGT2A3	a	t	0.9072	-0.2409	0.0407	3.20E-09	4290
rs4886584	15:79021464	LOC646934	a	g	0.3512	0.1689	0.0286	3.29E-09	3433
rs116532484	4:69710257	Intergenic (UGT2B10/UGT2A3)	a	g	0.0942	0.2117	0.0358	3.35E-09	4290
rs116293706	4:69724936	Intergenic (UGT2B10/UGT2A3)	t	c	0.0938	0.2157	0.0365	3.46E-09	4290
rs4886586	15:79026674	LOC646934	a	g	0.3619	0.179	0.0303	3.47E-09	3433
rs1825084	15:79023577	LOC646934	t	c	0.3232	0.1804	0.0308	4.63E-09	3433
rs1825085	15:79023578	LOC646934	a	g	0.6768	-0.1804	0.0308	4.64E-09	3433
rs4886583	15:79021445	LOC646934	a	g	0.3502	0.1671	0.0287	5.70E-09	3433
rs115838239	4:69821461	Intergenic (UGT2A3)	a	g	0.9037	-0.2331	0.0402	6.48E-09	4197
rs116605561	4:69739730	Intergenic (UGT2B10/UGT2A3)	a	c	0.89	-0.1922	0.0332	6.86E-09	4290
rs116052796	4:69741799	Intergenic (UGT2B10/UGT2A3)	t	g	0.11	0.192	0.0332	7.01E-09	4290
rs35474770	15:79074000	ADAMTS7	a	g	0.6124	-0.1462	0.0252	7.03E-09	4330
rs149163214	4:69743225	Intergenic (UGT2B10/UGT2A3)	a	c	0.8898	-0.1917	0.0331	7.16E-09	4290
rs114494748	4:69757183	Intergenic (UGT2B10/UGT2A3)	t	c	0.111	0.1901	0.0329	7.59E-09	4290
rs114826126	4:69766119	Intergenic (UGT2B10/UGT2A3)	a	t	0.8896	-0.1904	0.0331	8.88E-09	4290
rs115824708	4:69759022	Intergenic (UGT2B10/UGT2A3)	a	c	0.1107	0.1893	0.0329	8.90E-09	4290

rs115527001	4:69761239	Intergenic (UGT2B10/UGT2A3)	a	g	0.8893	-0.1892	0.033	9.44E-09	4290
rs114795848	4:69761311	Intergenic (UGT2B10/UGT2A3)	a	g	0.8893	-0.1892	0.033	9.46E-09	4290
rs115202858	4:69763587	Intergenic (UGT2B10/UGT2A3)	a	g	0.1107	0.1892	0.033	9.50E-09	4290
rs114742135	4:69766443	Intergenic (UGT2B10/UGT2A3)	a	t	0.8891	-0.1892	0.033	9.54E-09	4290
rs114237743	4:69770779	Intergenic (UGT2B10/UGT2A3)	t	g	0.1134	0.1883	0.0328	9.54E-09	4290
rs115835349	4:69766364	Intergenic (UGT2B10/UGT2A3)	t	c	0.1109	0.1892	0.033	9.59E-09	4290
rs114986615	4:69763910	Intergenic (UGT2B10/UGT2A3)	t	c	0.1108	0.1891	0.033	9.59E-09	4290
rs115479464	4:69718019	Intergenic (UGT2B10/UGT2A3)	t	g	0.8813	-0.1888	0.033	1.09E-08	4290
rs115389770	4:69772690	Intergenic (UGT2B10/UGT2A3)	a	g	0.887	-0.1903	0.0333	1.11E-08	4290
rs115485831	4:69642265	Intergenic (UGT2B15/UGT2B10)	a	t	0.9021	-0.2075	0.0363	1.13E-08	4290
rs115411305	4:69736764	Intergenic (UGT2B10/UGT2A3)	c	g	0.8855	-0.1849	0.0324	1.15E-08	4290
rs115069780	4:69755998	Intergenic (UGT2B10/UGT2A3)	a	g	0.1117	0.1876	0.0329	1.18E-08	4290
rs115826258	4:69771114	Intergenic (UGT2B10/UGT2A3)	t	c	0.113	0.1876	0.0329	1.21E-08	4290
rs115524872	4:69721246	Intergenic (UGT2B10/UGT2A3)	a	g	0.1139	0.188	0.033	1.27E-08	4290
rs116262380	4:69719134	Intergenic (UGT2B10/UGT2A3)	t	c	0.1157	0.1881	0.0331	1.28E-08	4290
rs115646318	4:69722947	Intergenic (UGT2B10/UGT2A3)	t	c	0.1136	0.1877	0.033	1.33E-08	4290
rs115756472	4:69729558	Intergenic (UGT2B10/UGT2A3)	a	g	0.8861	-0.1873	0.033	1.39E-08	4290
rs116691259	4:69730377	Intergenic (UGT2B10/UGT2A3)	a	g	0.1131	0.1874	0.033	1.42E-08	4290
rs116515246	4:69771309	Intergenic (UGT2B10/UGT2A3)	a	c	0.8867	-0.187	0.033	1.50E-08	4290
rs115600564	4:69771283	Intergenic (UGT2B10/UGT2A3)	c	g	0.8867	-0.187	0.033	1.50E-08	4290
rs114546260	4:69771354	Intergenic (UGT2B10/UGT2A3)	t	c	0.8867	-0.187	0.033	1.51E-08	4290
rs116603350	4:69771836	Intergenic (UGT2B10/UGT2A3)	a	g	0.8827	-0.1842	0.0326	1.54E-08	4197
rs114182442	4:69733034	Intergenic (UGT2B10/UGT2A3)	a	g	0.1088	0.1901	0.0336	1.58E-08	4290
rs115831974	4:69730266	Intergenic (UGT2B10/UGT2A3)	a	g	0.8909	-0.19	0.0336	1.61E-08	4290
rs115097835	4:69659738	Intergenic (UGT2B15/UGT2B10)	t	c	0.0983	0.1989	0.0352	1.63E-08	4197
rs11072792	15:78999911	Intergenic (LOC646934/ADAMTS7)	a	g	0.6092	-0.153	0.0272	1.80E-08	3433
rs28544432	15:78924538	CHRNB4	t	g	0.8279	0.2222	0.0395	1.80E-08	3433
rs114970731	4:69647536	Intergenic (UGT2B15/UGT2B10)	a	g	0.9027	-0.2001	0.0356	1.92E-08	4197
rs10163145	15:79075335	ADAMTS7	a	g	0.3994	0.1403	0.025	2.04E-08	4330
rs115988542	4:69709072	Intergenic (UGT2B10/UGT2A3)	a	c	0.8851	-0.182	0.0324	2.04E-08	4290
rs684513	15:78858400	CHRNA5	c	g	0.7582	0.1845	0.0329	2.05E-08	3433
rs115651349	4:69663632	Intergenic (UGT2B15/UGT2B10)	a	g	0.0965	0.2	0.0357	2.07E-08	4290
rs140205714	4:69664301	Intergenic (UGT2B15/UGT2B10)	t	g	0.9036	-0.2003	0.0357	2.08E-08	4290
rs114057562	4:69663476	Intergenic (UGT2B15/UGT2B10)	a	g	0.0965	0.1999	0.0357	2.09E-08	4290
rs115553258	4:69656777	Intergenic (UGT2B15/UGT2B10)	a	g	0.0978	0.1964	0.0351	2.14E-08	4290
rs36061084	15:79074253	ADAMTS7	a	g	0.4136	0.1441	0.0257	2.16E-08	4330
rs113444800	4:69661615	Intergenic (UGT2B15/UGT2B10)	a	g	0.903	-0.1985	0.0355	2.16E-08	4290
rs116001863	4:69658879	Intergenic (UGT2B15/UGT2B10)	t	c	0.902	-0.196	0.035	2.18E-08	4290
rs113999752	4:69659833	Intergenic (UGT2B15/UGT2B10)	a	c	0.098	0.1958	0.035	2.23E-08	4290
rs12441088	15:78928264	CHRNB4	t	g	0.7233	0.1709	0.0306	2.43E-08	3433
rs12442456	15:78751962	IREB2	t	g	0.1597	-0.2033	0.0365	2.63E-08	3433
rs1394371	15:78724469	Intergenic (IREB2)	t	c	0.2831	0.1259	0.0226	2.67E-08	4330
rs115762070	4:69771377	Intergenic (UGT2B10/UGT2A3)	a	t	0.1103	0.1865	0.0338	3.41E-08	4290
rs115501639	4:69771424	Intergenic (UGT2B10/UGT2A3)	t	c	0.1103	0.1865	0.0338	3.41E-08	4290
rs114727760	4:69771448	Intergenic (UGT2B10/UGT2A3)	c	g	0.8897	-0.1865	0.0338	3.42E-08	4290
rs4526984	15:78712119	Intergenic (IREB2)	a	t	0.2904	-0.1639	0.0297	3.47E-08	3433
rs951984	15:78720915	Intergenic (IREB2)	a	t	0.2749	0.1324	0.0241	4.21E-08	4330

Table S3. Descriptive characteristics of samples contributing to chromosome 4 replication analyses.

**Study**	***n***	**Sex (% male)**	**Age (years)**		**Cotinine (ng/ml)**[a]		**Medium**	**Method**	**Genotyping platform**	**Imputation**	
			Mean	SD	Mean	SD				Software	Reference
FinnTwin[b]	135	52.6	25.6	1.7	190.0	136.2	Serum	Mass spectrometry	Illumina CoreExome	IMPUTE 2	1000G phase 1 v3
FINRISK2007[b]	620	55.2	49.8	13.0	152.5	99.9	Serum	Mass spectrometry	Metabochip	N/A	N/A

[a] Cotinine mean and standard deviation values refer to raw values prior to standardisation (i.e., conversion to Z-scores). Further study details available in online methods. SD: standard deviation.

[b]Samples independent of the FinnTwin and FINRISK samples used in the discovery phase.

Table S4. Chromosome 4 signal replication results.

Replication sample	SNP	Chr	Gene	Position	LD with rs114612145	EA	EAF	*n*	Beta	SE	*p* value	*p* value in discovery
FinnTwin[a]	rs114612145 (rs77107237[b])	4	Intergenic (*UGT2B10*/*UGT2A3*)	69746647	N/A	G	0.07	135	0.56	0.23	**0.014**	5.89×10^{-10}
	rs144647471 (rs61750900[b])	4	*UGT2B10*	69681936	0.90	T	0.07	135	0.52	0.22	**0.020**	1.91×10^{-5}
FINRISK2007[a]	rs114866244 (rs6600854[b])	4	Intergenic (*UGT2B10*/*UGT2A3*)	69723767	0.88	C	0.06	620	0.32	0.12	**0.007**	2.12×10^{-9}

SNP: single nucleotide polymorphism; Chr: chromosome; LD: linkage disequilibrium; EA: effect allele; EAF: effect allele frequency; Beta: change in standard deviation of cotinine level per copy of the effect allele; SE: standard error. Position refers to base pair position in genome build hg19/GRCh37. LD with our top SNP in this region (rs114612145) calculated using https://caprica.genetics.kcl.ac.uk/~ilori/ld_calculator.php.

[a] Samples independent of the FinnTwin and FINRISK samples used in the discovery phase.
[b] Corresponding SNP rs IDs from build GRCh38.

Table S5. Summary of top SNPs identified from previous GWAS of self-reported smoking quantity and corresponding *p* values from cotinine GWAS.

**Author**	**Year**	***n***	**Chr**	**Gene**	**Top SNP**	***p* value**	***p* value from cotinine GWAS**
Thorgeirsson	2008	15,771	15	*CHRNA3*	rs1051730	6.00×10^{-20}	4.15×10^{-17}
Furberg	2010	73,853	15	*CHRNA3*	rs1051730	2.75×10^{-73}	4.15×10^{-17}
		73,853	10	*LOC100188947*	rs1329650	5.67×10^{-10}	0.89
		73,956	10	*LOC100188947*	rs1028936	1.29×10^{-9}	0.32
		73,853	19	*EGLN2*	rs3733829	1.04×10^{-8}	0.09
Thorgeirsson	2010	76,972	15	*CHRNA3*	rs1051730	2.40×10^{-69}	4.15×10^{-17}
		83,317	19	*CYP2A6*	rs4105144	2.20×10^{-12}	Not included
		84,956	8	*CHRNB3*	rs6474412	1.40×10^{-8}	0.08
Liu (Ox-GSK only)	2010	15,574	15	*CHRNA3*	rs1051730[*]	9.45×10^{-19}	4.15×10^{-17}
		15,574	15	*CHRNA5*	rs55853698[#]	1.31×10^{-16}	3.82×10^{-17}
		15,574	15	*CHRNA3*	rs6495308	3.30×10^{-10}	1.53×10^{-14}
David[¥]	2012	32,389	15	near *CHRNA5*	rs2036527	1.84×10^{-8}	4.01×10^{-17}

*SNP with lowest *p*-value from HapMap (release 22) based analyses in Ox-GSK.
#SNP with lowest *p*-value from 1000 Genomes (Pilot 1) based analyses in Ox-GSK.
¥ African American sample
